# Donor IL-17 receptor A regulates LPS-potentiated acute and chronic murine lung allograft rejection

**DOI:** 10.1172/jci.insight.158002

**Published:** 2023-11-08

**Authors:** Tatsuaki Watanabe, Stephen C. Juvet, Gregory Berra, Jan Havlin, Wenshan Zhong, Kristen Boonstra, Tina Daigneault, Miho Horie, Chihiro Konoeda, Grace Teskey, Zehong Guan, David M. Hwang, Mingyao Liu, Shaf Keshavjee, Tereza Martinu

**Affiliations:** 1 Latner Thoracic Research Laboratories, University Health Network, Toronto, Ontario, Canada.; 2 Department of Thoracic Surgery, Institute of Development, Aging and Cancer, Tohoku University, Sendai, Japan.; 3 Toronto Lung Transplant Program, Ajmera Transplant Center, University Health Network, Toronto, Ontario, Canada.; 4 Division of Respirology, Department of Medicine, University of Toronto, Toronto, Ontario, Canada.; 5 Joint Department of Medical Imaging and; 6 Department of Pathology, University Health Network, Toronto, Ontario, Canada.; 7 Department of Laboratory Medicine and Molecular Diagnostics, Sunnybrook Health Sciences Centre, Toronto, Ontario, Canada.; 8 Division of Thoracic Surgery, Department of Surgery, University of Toronto, Toronto, Ontario, Canada.

**Keywords:** Pulmonology, Transplantation, Adaptive immunity, Fibrosis, Innate immunity

## Abstract

Chronic lung allograft dysfunction (CLAD) is a major complication after lung transplantation that results from a complex interplay of innate inflammatory and alloimmune factors, culminating in parenchymal and/or obliterative airway fibrosis. Excessive IL-17A signaling and chronic inflammation have been recognized as key factors in these pathological processes. Herein, we developed a model of repeated airway inflammation in mouse minor alloantigen-mismatched single-lung transplantation. Repeated intratracheal LPS instillations augmented pulmonary IL-17A expression. LPS also increased acute rejection, airway epithelial damage, and obliterative airway fibrosis, similar to human explanted lung allografts with antecedent episodes of airway infection. We then investigated the role of donor and recipient IL-17 receptor A (IL-17RA) in this context. Donor IL-17RA deficiency significantly attenuated acute rejection and CLAD features, whereas recipient IL-17RA deficiency only slightly reduced airway obliteration in LPS allografts. IL-17RA immunofluorescence positive staining was greater in human CLAD lungs compared with control human lung specimens, with localization to fibroblasts and myofibroblasts, which was also seen in mouse LPS allografts. Taken together, repeated airway inflammation after lung transplantation caused local airway epithelial damage, with persistent elevation of IL-17A and IL-17RA expression and particular involvement of IL-17RA on donor structural cells in development of fibrosis.

## Introduction

Lung transplantation is an established life-saving therapy for patients with end-stage lung disease. However, median survival after lung transplantation is the lowest among solid-organ transplants and remains at only 5–6 years (1) because of chronic lung allograft dysfunction (CLAD), the manifestation of chronic rejection. CLAD is clinically characterized by irreversible airway and lung parenchymal fibrosis and is caused primarily by an antidonor alloimmune response. Despite ongoing refinement of immunosupressive strategies, CLAD remains a progressive and untreatable condition (2).

CLAD is clinically classified in several subtypes, of which the most common is bronchiolitis obliterans syndrome (BOS), characterized primarily by airflow obstruction. The pathology associated with BOS includes bronchiolitis obliterans (BO), which causes obliteration of small airways with fibrous tissue (3). A smaller subset of patients has other types of CLAD, such as restrictive allograft syndrome (RAS), manifesting as lung restriction with parenchymal fibrosis on radiologic imaging and associated with pleuroparenchymal fibroelastosis (PPFE) on histology (4).

Different from other solid organs, lungs continuously communicate with the external environment via the airways. Lung allografts are exposed to pathogens, pollutants, altered microbiome, and aspiration of oropharyngeal contents (5–8). These innate immune stimuli can activate injurious adaptive immune mechanisms (9). In fact, airway damage via innate immune stimuli has been proposed as an underlying reason for the relatively poor outcomes after lung transplantation (10). However, how these stimuli augment rejection and result in lung remodeling and impairment is incompletely understood.

Several small-animal models have been used for CLAD research. Although tracheal transplant models have been used widely for investigation of BO (11–13), being less technically demanding and more reproducible, they have fundamental limitations such as lack of vascularization, airway ventilation, exposure to external environment, and exposure to a pulmonary immune milieu. Vascularized orthotopic mouse lung transplantation models overcome these specific limitations and allow investigation of immunological mechanisms relevant to clinical lung transplantation (14). Major histocompatibility–mismatched mouse lung transplantation shows severe fulminant acute rejection and lung destruction (15). Experimental models with gradual parenchymal and/or airway damage and fibrosis, replicating human CLAD pathology without complete destruction of the lung allograft, are ideal platforms for CLAD research. Fan et al. revealed that a minor alloantigen-mismatched orthotopic lung transplantation model using C57BL/10 (B10, carrying H-2^b^) to C57BL/6 (B6, carrying H-2^b^) mice generates pathological features of CLAD (16). We also observed that this model exhibited a spectrum of allograft pathology with mild acute rejection and variable airway and parenchymal fibrosis (17). We further noticed that the incidence and severity of allograft fibrosis in this model decreased over time, similar to findings reported by Yamada and colleagues as well as Guo and colleagues, possibly due to increasing surgical experience, subtle changes in surgical techniques, and variability in microbiome, vendor, or housing conditions (18, 19). Our recent work shows that prolonged graft storage enhanced chronic airway and parenchymal fibrosis in this model (20), suggesting that donor lung injury contributes to the development of CLAD.

Recurrent infections and chronic inflammation after transplantation also contribute to the pathogenesis of CLAD. LPS, a component of the outer membrane of gram-negative bacteria and a ligand of TLR4, is highly immunogenic and thought to drive inflammation during infections (5). In a rat transplantation model, intratracheal LPS administration resulted in pathological changes similar to those seen in human CLAD and BO (21, 22). Activation of alloimmunity by LPS-induced inflammation can induce pulmonary injury and break tolerance after allogeneic BM transplantation (23). Moreover, airway administration of Pam_3_ Cys_4_, an agonist of TLR2, to donor lungs prevented lung graft acceptance through a process mediated by recipient-derived monocytes in a major alloantigen-mismatched mouse lung transplantation model (24).

IL-17A has a critical role in mucosal host defenses. However, excessive and prolonged IL-17A signaling activation is associated with lung damage and remodeling (25). Accumulating data suggest that IL-17A contributes to the development of chronic lung rejection in humans and mice (16, 26–31). Several types of cells in the lung can produce IL-17A, including IL-17A–producing CD4^+^ Th lymphocytes (Th17), NK cells, γδ T cells, αβ T cells, and group 3 innate lymphoid cells (32). The downstream signaling of IL-17A requires the IL-17 receptor A (IL-17RA) and IL-17RC heterodimer (33). IL-17RA can be expressed in a variety of cell types, including fibroblasts, epithelial cells, B cells, T cells, and eosinophils (34, 35). The relative expression and contribution of recipient and donor IL-17RA in lung transplantation remain incompletely studied.

We hypothesized that chronic airway inflammation–induced tissue injury enhances alloimmunity and contributes to the development of acute rejection and CLAD. Additionally, we postulated that IL-17RA on both recipient and donor cells is involved in this pathogenic process.

In this study, we first characterized airway epithelial and fibrotic changes in patients who developed CLAD after repeated infectious episodes. We then showed that repeated airway stimulation with intratracheal LPS instillations, mimicking repeated infections, in a mouse lung transplantation model augmented neutrophil and T cell recruitment, airway damage, and airway fibrosis in the context of alloimmunity; it also promoted persistent elevation of IL-17A and upregulation of IL-17RA, expressed on fibroblasts, myofibroblasts, and some immune cells. This was consistent with enhanced IL-17RA staining in the explanted human CLAD lungs with prior airway infection episodes. We then determined, using IL-17RA–KO mice, that IL-17RA on donor cells, more so than on recipient cells, is necessary for the LPS-mediated augmentation of acute rejection and airway fibrosis.

## Results

### Epithelial changes and destruction in human lung allografts with BO.

**Figure 1:**
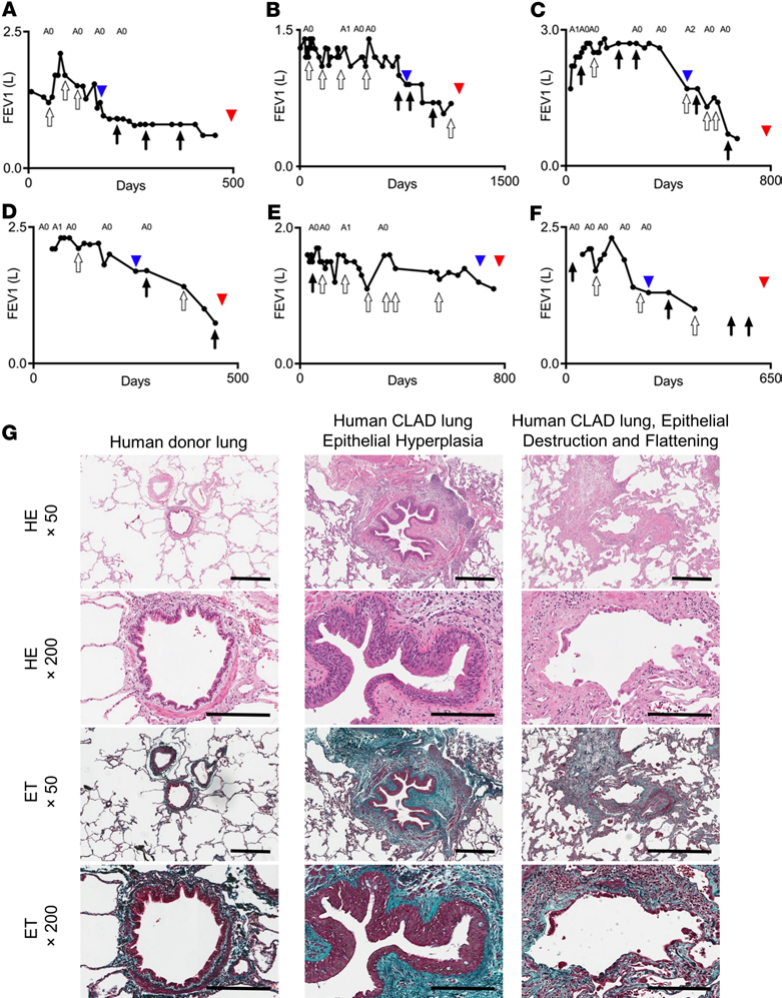
CLAD lungs with antecedent infectious episodes. Human CLAD allografts from patients with repeated pulmonary infection episodes after transplant. (**A**–**F**) Trajectories of forced expiratory volume in 1 second (FEV_1_) over time, of lung transplant recipients with recurrent episodes of pulmonary infections followed by CLAD development. Black arrows indicate gram-negative bacterial infection. White arrows indicate infection with bacteria other than gram-negative bacteria. A blue arrowhead indicates the onset of CLAD. A red arrowhead indicates retransplantation. Acute rejection A-grades, based on transbronchial biopsies, are indicated above the graphs. (**A**) Case 1 in Supplemental Table 1. (**B**) Case 2 in Supplemental Table 1. (**C**) Case 3 in Supplemental Table 1. (**D**) Case 4 in Supplemental Table 1. (**E**) Case 5 in Supplemental Table 1. (**F**) Case 6 in Supplemental Table 1. (**G**) Representative images of airway pathology from explanted CLAD lungs compared with a human donor lung as control. Left: Donor lung sample obtained at volume reduction during transplantation showed normal airway epithelium. Middle: Explanted CLAD lung (Case A) showed airway epithelial hyperplasia. Right: Explanted CLAD lungs (Case F) showed airway epithelial destruction and epithelial flattening. Scale bar: 200 μm. ET, Elastic Trichrome.

We retrospectively reviewed human lung allografts that were explanted during redo transplantation for CLAD. We identified 6 patients who had 2 or more episodes of airway infection prior to CLAD onset (36) (Supplemental Table 1; supplemental material available online with this article; https://doi.org/10.1172/jci.insight.158002DS1). All patients had at least 1 gram-negative infection (pathogens bolded in Supplemental Table 1). These patients experienced a gradual decline in their lung function after the airway infections (Figure 1, A–F), although other risk factors were also identified (Supplemental Table 1). The explanted lung specimens exhibited histologic BO with findings including epithelial hyperplasia, flattening, and destruction (Figure 1G). The subsequent experiments aimed to model these findings in mice.

### Repeated airway LPS administration resulted in periairway fibrosis and airway obliteration in mouse lung allografts.

**Figure 2:**
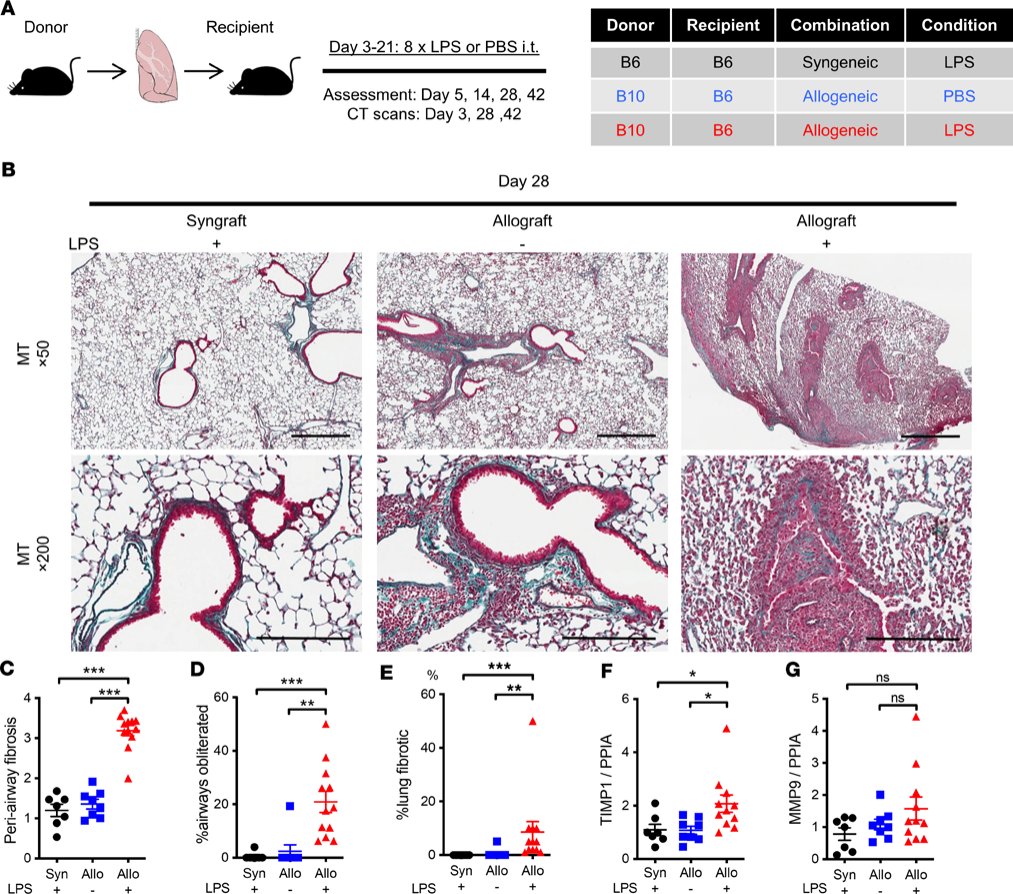
Repeated LPS exposures result in augmentation of chronic rejection pathology. B6 recipient mice received a single left lung transplant from B6 or B10 donor mice, followed by repeated intratracheal LPS versus PBS exposures. (**A**) Schema of the experiment. Each group received 8 doses of intratracheal LPS (5 μg in 50 μL PBS) or PBS (50 μL) on serial (3/week) postoperative days. Experimental groups are as follows: syngrafts exposed to LPS (LPS syngrafts), allografts exposed to PBS (PBS allografts), and allografts exposed to LPS (LPS allografts). The grafts were assessed on days 5, 14, 28, and 42. (**B**) Representative images of Masson’s trichrome (MT) staining on day 28 after lung transplantation. Upper scale bar: 500 μm. Lower scale bar: 200 μm. Left: LPS syngrafts. Middle: PBS allograft. Right: LPS allograft. LPS syngraft and PBS allograft showed no obvious fibrosis. LPS allografts showed augmented periairway fibrosis and obliterated airways. (**C**–**E**) Fibrotic change scores of the lung grafts (*n* = 7–12 per group). Data are shown as mean ± SEM. (**C**) Periairway fibrosis score. (**D**) Percentage of obliterated airways. (**E**) Percentage of lung parenchymal fibrosis. (**F**) Lung graft TIMP1 transcript normalized to peptidylprolyl isomerase A (PPIA) housekeeping gene. (**G**) Lung graft MMP9 transcript normalized to PPIA. Kruskal-Wallis test. *P < 0.05; **P < 0.01; ***P < 0.001.

To determine the relationship between posttransplant chronic innate immune stimulation and airway fibrosis and obliteration, a minor alloantigen-mismatched mouse lung transplantation system was used. We exposed allografts and syngrafts to repeated posttransplant intratracheal instillations of LPS or phosphate-buffered saline (PBS) (Figure 2A). At day 28, consistent with our previous observations (20, 37) and similar to published data (18), PBS-treated allografts had findings of acute rejection histology and minimal fibrotic changes in the airways (Figure 2B, middle panels), compared with almost normal-appearing LPS-challenged syngrafts (Figure 2B, left panels). In contrast, LPS-challenged allografts showed periairway and obliterative airway fibrosis (Figure 2B, right panels). Average periairway fibrosis scores, obliterated airway percentages, and lung parenchymal fibrosis area percentages were all significantly higher in LPS-challenged allografts compared with the other 2 groups by blinded histological grading (Figure 2, C–E) as well as by automated Trichrome color quantification by HALO software (Supplemental Figure 1, A and B). We then assessed tissue remodeling factors such as tissue inhibitor of metalloproteinases-1 (TIMP1) and matrix metalloproteinase-9 (MMP9), previously shown to be associated with airway obliteration in transplant models (38, 39). TIMP1 transcripts were significantly increased in LPS allografts compared with controls (Figure 2F). A subset of LPS allografts demonstrated increased MMP9, without a statistically significant difference between the groups (Figure 2G). Fibrotic remodeling, based on histological fibrosis scores and TIMP1 elevation, was persistently observed in LPS allografts at day 42 (Supplemental Figure 1, C–G). In contrast, MMP9 trended lower at this later time point, consistent with some reports of an inverse relationship between MMP9 and fibrosis (Supplemental Figure 1H) (40, 41). These observations indicated that recurrent airway inflammation enhanced alloimmune-dependent airway fibrosis and airway obliteration — and, to a lesser degree, parenchymal fibrosis — in this mouse lung transplant model.

**Figure 3:**
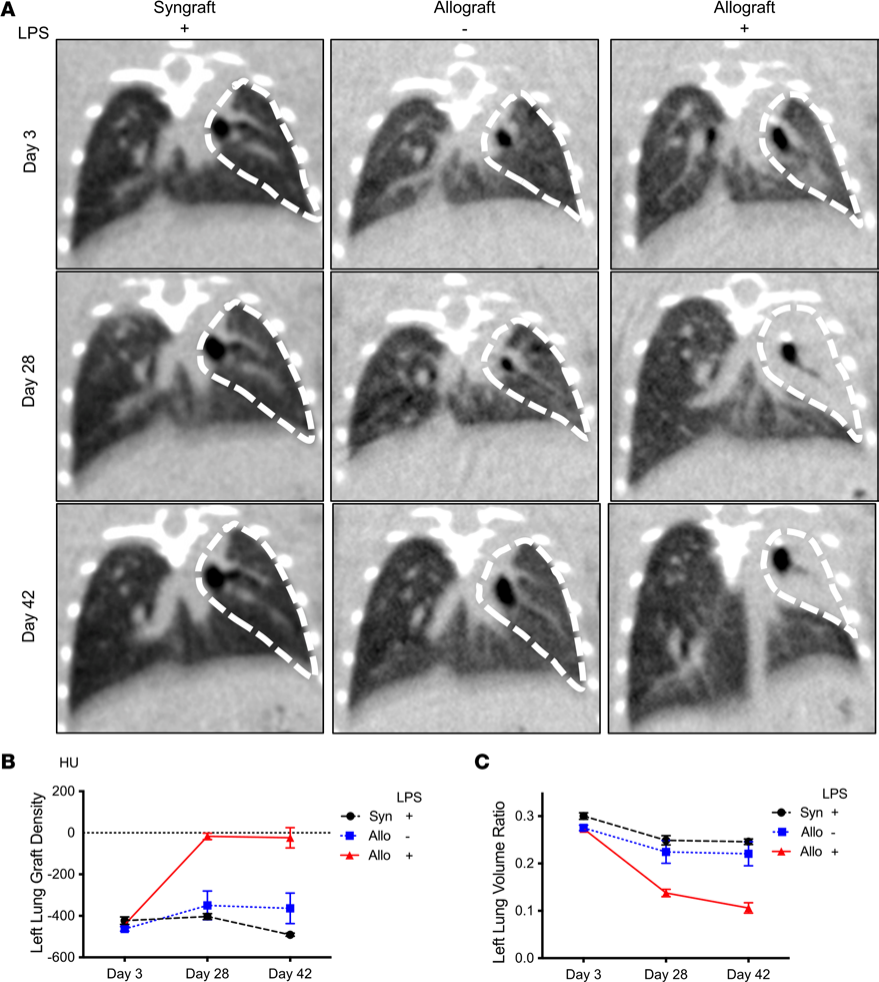
Repeated LPS exposures result in lung volume reduction and an increase in lung density. (**A**) Representative pictures of CT scans. (**B** and **C**) Quantification of μCT data (*n* = 6–8). (**B**) Left lung graft density calculated from the μCT scan data. Left lung graft density was significantly higher in LPS allografts than in LPS syngrafts and PBS allografts on day 28 (*P* < 0.0001) and day 42 (*P* < 0.0001). (**C**) Left lung graft volume ratio was calculated as left lung volume divided by right lung volume from the μCT scan data. Left lung volume ratio was significantly lower in LPS allografts compared with LPS syngrafts and PBS allografts on days 28 (*P* < 0.0001) and 42 (*P* < 0.0001). Data are shown as mean ± SEM. CT data were compared by 2-way ANOVA.

To further characterize time-dependent changes in LPS-stimulated allografts, we assessed longitudinal radiographic changes of the graft by μCT on days 3, 28, and 42 after lung transplantation. LPS syngrafts and PBS allografts showed clear lung fields and stable lung volume ratios throughout the period (Figure 3, A–C). Lung fields of LPS allografts were clear on day 3; however, in contrast to control groups, they showed consolidation in left lung grafts on day 28 and day 42 (Figure 3A), and their volume ratio gradually decreased (Figure 3B). Lung graft density of LPS allografts was significantly higher than LPS syngrafts and PBS allografts (Figure 3C). These results suggest that, even after cessation of LPS stimulation, lung damage persisted specifically in LPS-exposed allografts.

### Repeated LPS administration led to epithelial abnormalities in an alloimmune-dependent manner.

**Figure 4:**
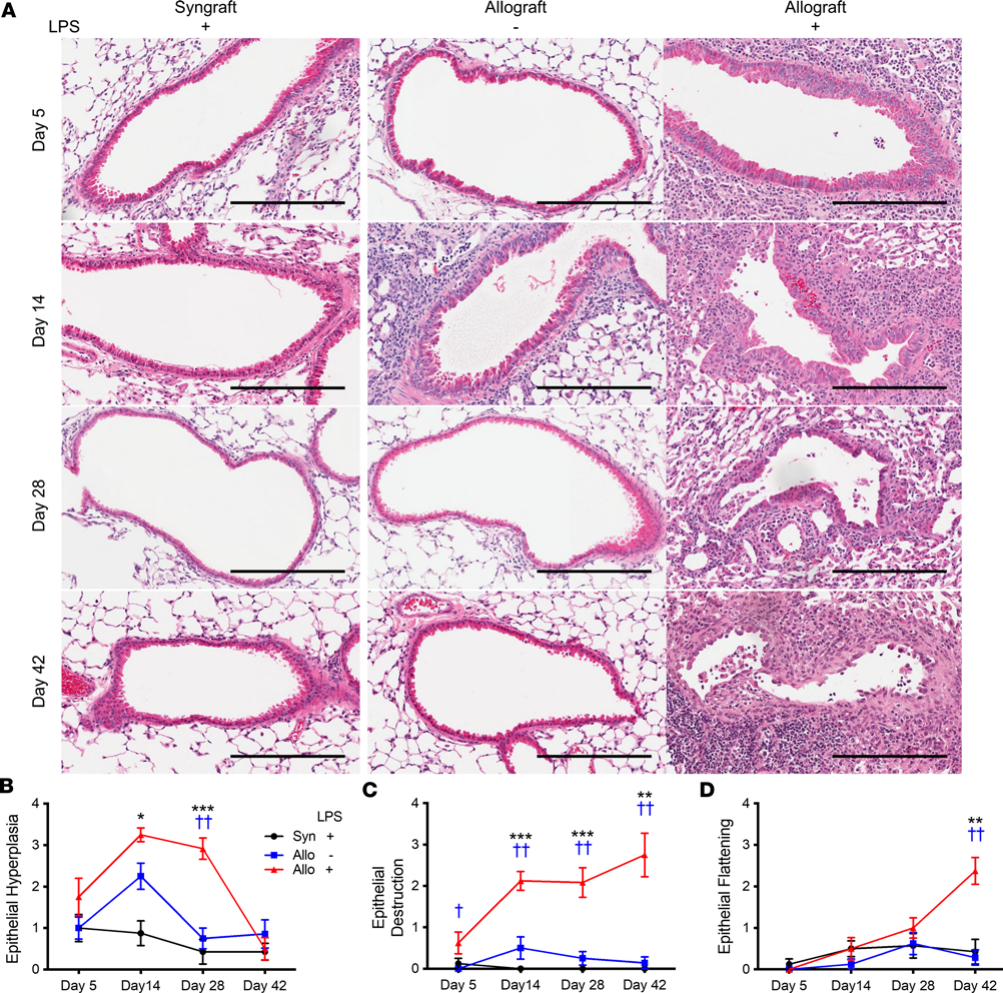
Repeated LPS exposures result in alloimmune-dependent epithelial changes. B6 recipient mice received a single left lung transplant from B6 or B10 donor mice, followed by repeated intratracheal LPS versus PBS exposures. (**A**) Representative images of the airways in the lung graft after transplantation on days 5, 14, 28, and 42. H&E staining. Scale bar: 200 μm. Left: LPS syngrafts. Middle: PBS allografts. Right: LPS allografts. LPS syngrafts did not show obvious epithelial injury. Airway epithelial hyperplasia was observed in LPS allografts on day 14, and it resolved by day 28 and 42. Airway epithelial destruction and flattening were not observed in PBS allografts. LPS allografts showed hyperplastic epithelium and destruction on days 14 and 28. On day 42, epithelial destruction and epithelial flattening were observed in LPS allografts. (**B**–**D**) Airway epithelial scores in the lung grafts (*n* = 7–12 per group, mean ± SEM). (**B**) Epithelial hyperplasia graded from 0 to 4. (**C**) Epithelial destruction graded from 0 to 4. (**D**) Epithelial flattening graded from 0 to 4. Kruskal-Wallis test. LPS syngrafts versus LPS allografts, *P < 0.05; **P < 0.01; ***P < 0.001. PBS allografts versus LPS allografts, ^†^*P* < 0.05; ^††^*P* < 0.01.

Airway epithelial injury is an important feature in tracheal transplant models of chronic rejection (42, 43) and occurs in human BO (Figure 1G). We therefore analyzed the airway epithelium in our experimental model. In LPS syngrafts, the airway epithelium was maintained with a near-normal appearance at all time points (Figure 4A). PBS allografts showed minimal airway destruction and flattening on day 14, with mild hyperplasia, which was almost completely resolved by days 28 and 42 (Figure 4, A–D). In contrast, pronounced epithelial hyperplasia and epithelial destruction were observed in LPS allografts on days 14 and 28. Epithelial destruction continued to increase until day 42, despite cessation of LPS administration after day 21 (Figure 4, A–C). Moreover, LPS allografts exhibited significant epithelial flattening at day 42 (Figure 4, A and D). These airway changes in LPS allografts were similar to those seen in human explanted CLAD lungs (Figure 1G).

**Figure 5:**
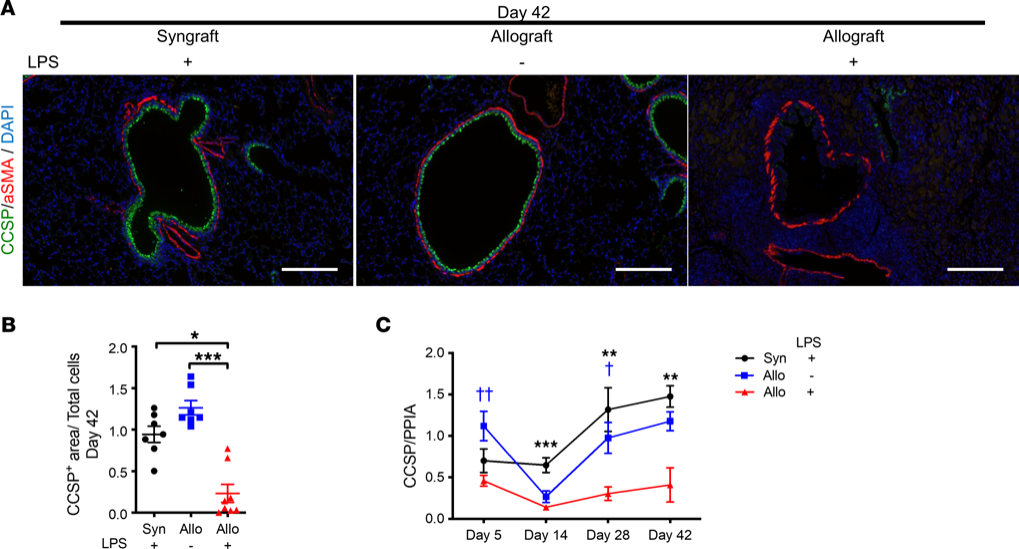
Repeated LPS exposures result in alloimmune-dependent CCSP reduction. (**A**) Representative immunofluorescence of α-SMA (red), CCSP (green), and DAPI (blue) of the grafts on day 42 after transplantation (*n* = 7–8). Scale bar: 200 μm. Left: LPS syngraft. Middle: PBS allograft. Right: LPS allograft. (**B**) Ratio of CCSP^+^ area normalized to total cell numbers. (**C**) Relative expression of CCSP transcripts normalized to peptidylprolyl isomerase A (PPIA) housekeeping gene on days 5, 14, 28, and 42 after transplantation (*n* = 7–12). Data are shown as mean ± SEM. Kruskal-Wallis test. LPS syngrafts versus LPS allografts, *P < 0.05; **P < 0.01; ***P < 0.001. PBS allografts versus LPS allografts, ^†^*P* < 0.05; ^††^*P* < 0.01.

Club cell secretory protein–expressing (CCSP-expressing) cells are critical for lung regeneration (44), and downregulation of CCSP has been associated with CLAD and BO (45–49). In our model, CCSP^+^ cells were identified in the airways of LPS syngrafts and PBS allografts but were significantly reduced in LPS allograft airways at day 42 (Figure 5, A and B). CCSP mRNA levels in LPS allografts were significantly lower than in LPS syngrafts on days 14, 28, and 42 and in PBS allografts on days 5 and 28 (Figure 5C). Taken together, airway innate immune stimulation led to epithelial damage and reduction in CCSP^+^ cells in an alloimmune-dependent manner.

### Repeated LPS administration enhanced acute rejection pathology with T cell recruitment to the allografts.

**Figure 6:**
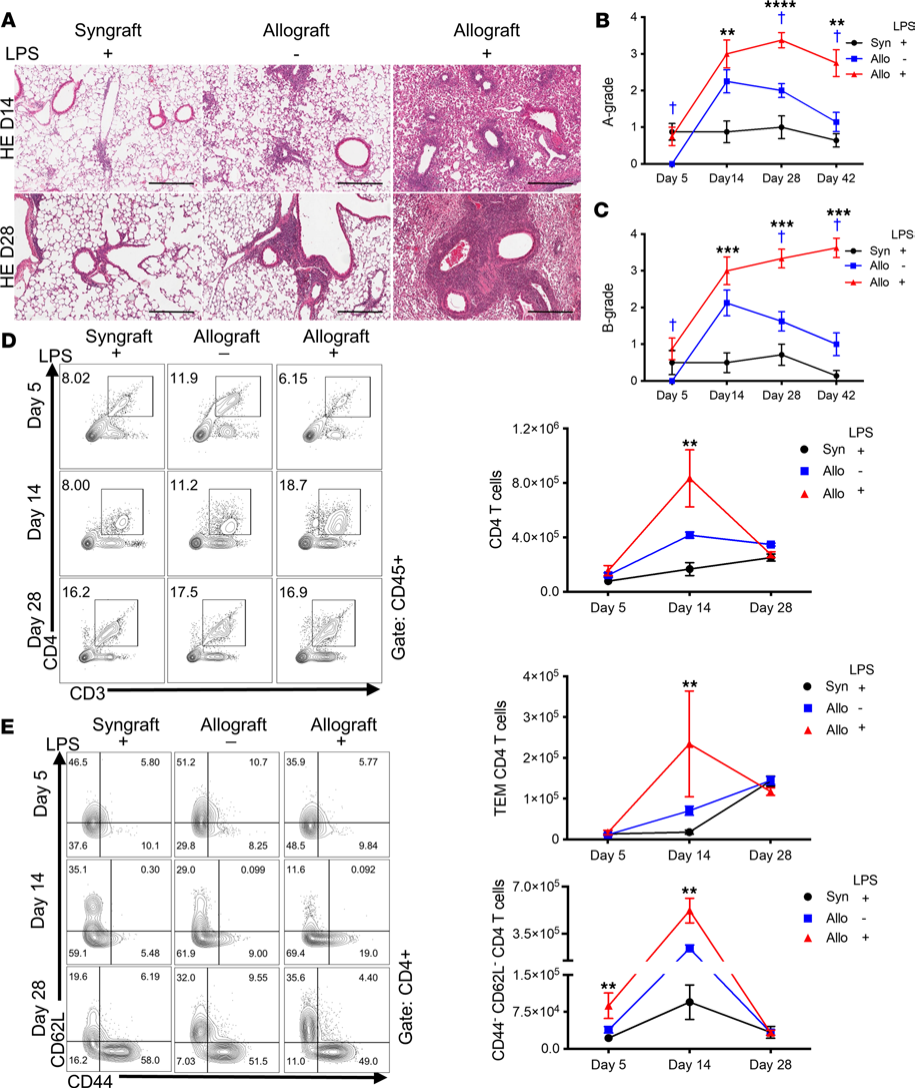
Repeated LPS exposures augment adaptive immune responses. B6 recipient mice received a single left lung transplant from B6 or B10 donor mice, followed by repeated intratracheal LPS versus PBS exposures. (**A**) Representative pictures of H&E staining on days 14 and 28 (Scale bar: 300 μm). Left: LPS syngraft. Middle: PBS allograft. Right: LPS allograft. Upper images are 14 days after lung transplantation. Bottom images are 28 days after lung transplantation. (**B** and **C**) Perivascular and periairway lymphocyte infiltration histological rejection scores of lung grafts on days 5, 14, 28, and 42 (*n* = 7–12 per group). Data are shown as mean ± SEM). (**B**) A-grade. (**C**) B-grade. Kruskal-Wallis test. LPS syngrafts versus LPS allografts, **P < 0.01; ***P < 0.001; ****P < 0.0001. PBS allografts versus LPS allografts, ^†^*P* < 0.05. (**D** and **E**) CD4^+^ T cells and their subsets by lung cell flow cytometry on days 5, 14, and 28 (*n* = 3–4 per group). Data are shown as mean ± SEM). Data for each time point were obtained from different animals. (**D**) Total CD4^+^ T cells in lung grafts. (**E**) Effector memory CD4^+^ T cells (CD44^+^CD62L^–^CD4^+^ T cells, TEM) and CD44^–^CD62L^–^CD4^+^ T cells. Kruskal-Wallis test. LPS syngrafts versus LPS allografts, **P < 0.01.

Acute cellular rejection has been identified as a consistent risk factor for CLAD in human lung transplant recipients (50, 51). Therefore, we assessed acute rejection pathology and the presence and state of T cells in the mouse lung grafts. At days 28 and 42, LPS allografts exhibited significantly higher perivascular (A-grade) and peribronchial/peribronchiolar (B-grade) acute rejection histology scores than LPS syngrafts and PBS allografts (Figure 6, A–C). On day 14, LPS allografts contained more CD4^+^ T cells (Figure 6D), with increased subpopulations of CD44^+^CD62L^–^ (effector memory) and CD44^–^CD62L^–^ (stunted or possibly recently activated; ref. 52) CD4^+^ T cells (Figure 6E and Supplemental Figure 2, A and B) and CD8^+^ T cells (Supplemental Figure 3), compared with LPS syngrafts, with a modest nonsignificant increase compared with PBS allografts.

### Repeated airway LPS administration enhanced acute lung injury, neutrophils, and IL-17A in lung allografts.

**Figure 7:**
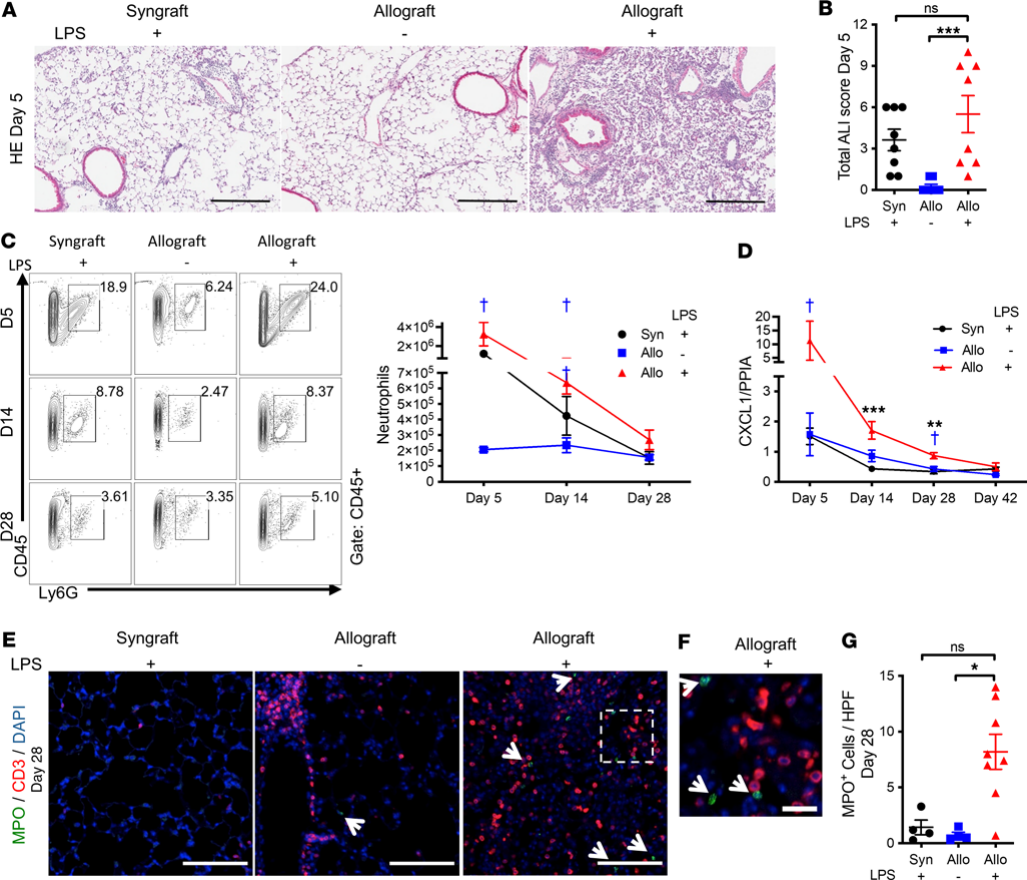
Repeated LPS exposures result in increased acute lung injury, neutrophils, and CXCL1. B6 recipient mice received a single left lung transplant from B6 or B10 donor mice, followed by repeated intratracheal LPS versus PBS exposures. (**A**) Representative H&E staining of lung grafts on day 5. Scale bar: 300 μm. Left: LPS syngraft. Middle: PBS allograft. Right: LPS allograft. (**B**) Total acute lung injury on day 5 graded from 0 to 12 (*n* = 8 per group). Kruskal-Wallis test. ***P < 0.001. (**C**) Neutrophils in lung grafts on days 5, 14, and 28 by lung cell flow cytometry (*n* = 3–4 per group). Data are shown as mean ± SEM). Data from each time point were obtained from different animals. ^†^*P* < 0.05. (**D**) CXCL1 transcript normalized to PPIA in the grafts over time. (**E** and **F**) Representative pictures of immunofluorescence for CD3 and MPO on day 28. (**E**) Left: LPS syngraft. Middle: PBS allograft. Right: LPS allograft. green, MPO; red, CD3; blue, DAPI. Arrows indicate MPO^+^ cells. Scale bar: 100 μm. (**F**) A magnified image of LPS allograft. Scale bar: 20 μm. (**G**) MPO^+^ cells in the grafts. The numbers are the sum of the MPO^+^ cells in 10 randomly selected fields (*n* = 4–8). Kruskal-Wallis test. *P < 0.05.

**Figure 8:**
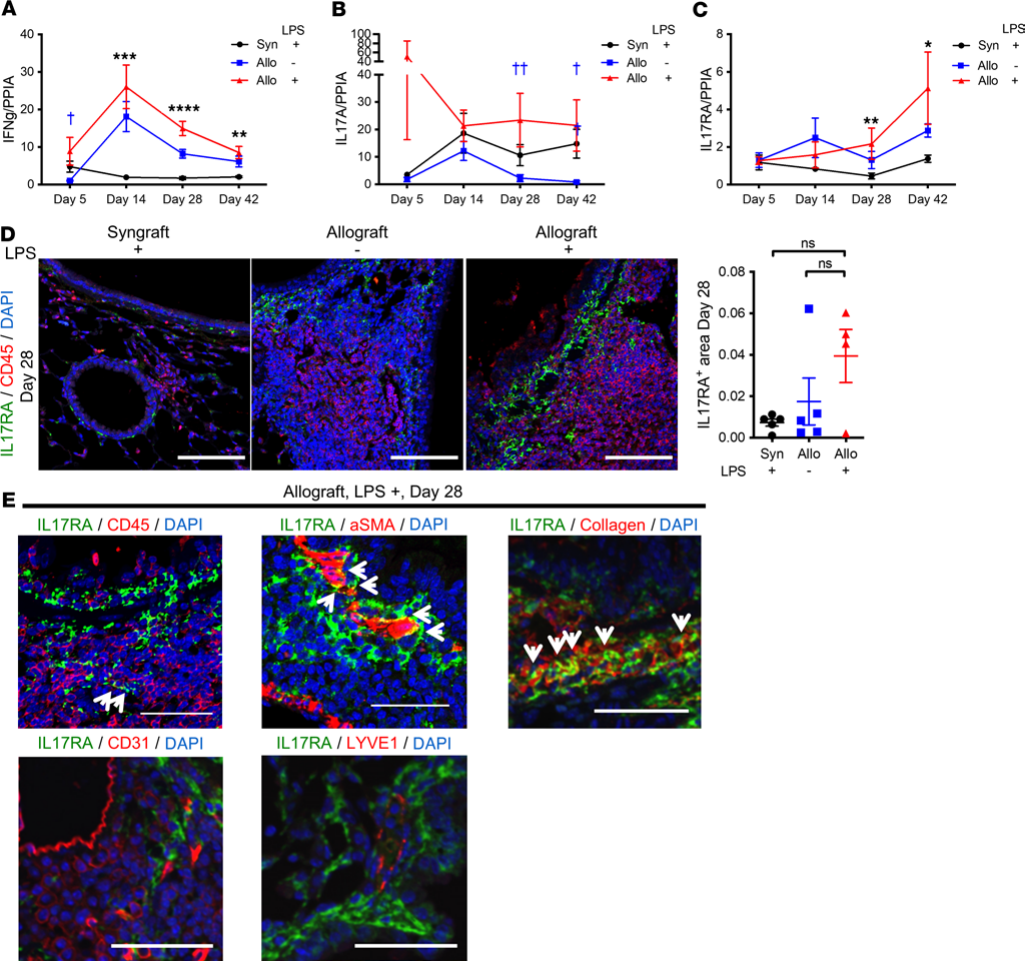
Repeated LPS exposures result in increased IL-17A and IL-17RA in the graft. B6 recipient mice received a single left lung transplant from B6 or B10 donor mice, followed by repeated intratracheal LPS versus PBS exposures. (**A**–**C**) Relative expression of cardinal genes. Transcripts were normalized to PPIA (*n* = 7–12 per group). Data are shown as mean ± SEM. Each time point represents different animals. (**A**) IFN-γ transcript expression in the lung grafts. (**B**) IL-17A transcript expression in lung grafts. (**C**) IL-17RA transcript expression in lung grafts. (**D**) Representative pictures of immunofluorescence for IL-17RA and CD45 on day 28. Left: LPS syngraft. Middle: PBS allograft. Right: LPS allograft. Red, CD45; green, IL-17RA; blue, DAPI. Scale bar: 100 μm. Kruskal-Wallis test. LPS syngrafts versus LPS allografts, *P < 0.05; **P < 0.01; ***P < 0.001; ****P < 0.0001. PBS allografts versus LPS allografts, ^†^*P* < 0.05; ^††^*P* < 0.01. (**E**) Representative pictures of immunofluorescence for IL-17RA and CD45, α-SMA, Collagen, CD31, or LYVE1. Arrows indicate dual staining of the antibodies. Scale bar: 50 μm. Upper right panel: IL-17RA and CD45. Green, IL-17RA; red, CD45; blue, DAPI. This image is a part of Figure 7D right panel. Upper middle panel: IL-17RA and α-SMA. Green, IL-17RA; red, α-SMA; blue, DAPI. Upper right panel: IL-17RA and Collagen. Green, IL-17RA; red, Collagen; blue, DAPI. Lower left panel: IL-17RA and CD31. Green, IL-17RA; red, CD31; blue, DAPI. Lower right panel: IL-17RA and LYVE1. Green, IL-17RA; red, LYVE1; blue, DAPI.

Acute lung injury pathology has been identified as a risk factor for CLAD (53). Additionally, intragraft neutrophils have been implicated in development of acute rejection and break in established tolerance in a mouse lung transplant model (54, 55). Neutrophils can function as antigen-presenting cells to promote T cell responses (56). We therefore assessed these phenomena in our model. Significant acute lung injury with leukocyte infiltration was seen in LPS allografts, but not PBS allografts, on day 5 after transplant (Figure 7, A and B). Enhanced neutrophil recruitment was identified in LPS allografts compared with PBS allografts on days 5 and 14 after transplant (Figure 7C). On day 28, by flow cytometry, LPS allografts still showed a trend toward increased neutrophils compared with both control groups (Figure 7C and Supplemental Figure 3B); this difference was found to be significant when a larger number of day 28 paraffin-embedded samples were analyzed by myeloperoxidase (MPO) immunofluorescence staining (Figure 7, E–G). Increased T cells were also identified by CD3 immunostaining in LPS allografts on day 28. We therefore examined the gene expression of cytokines that are known to be related to neutrophil and T cell recruitment, and previously linked to CLAD: CXCL1, IL-17A, and IFN-γ (26, 28, 57). Transcripts for CXCL1, an important neutrophil chemoattractant, were significantly higher in LPS allografts than in LPS syngrafts or PBS allografts up to day 28 (Figure 7D), 7 days after the cessation of airway LPS administration. IFN-γ transcripts in LPS allografts were significantly higher than in LPS syngrafts but not statistically higher compared with PBS allografts (Figure 8A). In contrast, LPS allografts showed higher IL-17A transcript expression on days 28 and 42 compared with the other 2 groups (Figure 8B). The augmented IL-17A expression in LPS allografts in the later phase prompted us to assess expression of the IL-17A receptor, IL-17RA. IL-17RA transcript expression in LPS allografts was also significantly higher than LPS syngrafts on days 28 and 42, with a trend toward higher expression compared with PBS allografts (Figure 8C).

In addition, immunofluorescence staining of IL-17RA shows a trend toward higher expression in LPS allografts compared with LPS syngrafts and PBS allografts (Figure 8D). IL-17RA staining was not visible within airway or alveolar epithelial cells. In LPS allografts, α-SMA and collagen, and minimally also CD45, costained with IL-17RA (Figure 8E and Supplemental Figure 4, A–C). CD31 and LYVE1 did not costain with IL-17RA (Supplemental Figure 4, D and E). These results suggest that fibroblasts and myofibroblasts may be targets of IL-17A in this model, with leukocytes being less-important targets.

Since the augmented and persistent airway epithelial damage, acute rejection, fibrosis, and IL-17A and IL-17RA transcript upregulation were dependent on both alloimmunity and repeated airway stimulation with LPS, we set out to examine the involvement of IL-17RA in our experimental model. IL-17RA on donor cells or on recipient cells was evaluated separately by conducting transplant experiments using *Il17ra^–/–^* donor or recipient mice, with and without LPS instillations. We primarily focused on day 28 endpoints, given their relevance to CLAD.

### IL-17RA deficiency on recipient cells has minimal effects on allograft pathology in the context of LPS exposures.

**Figure 9:**
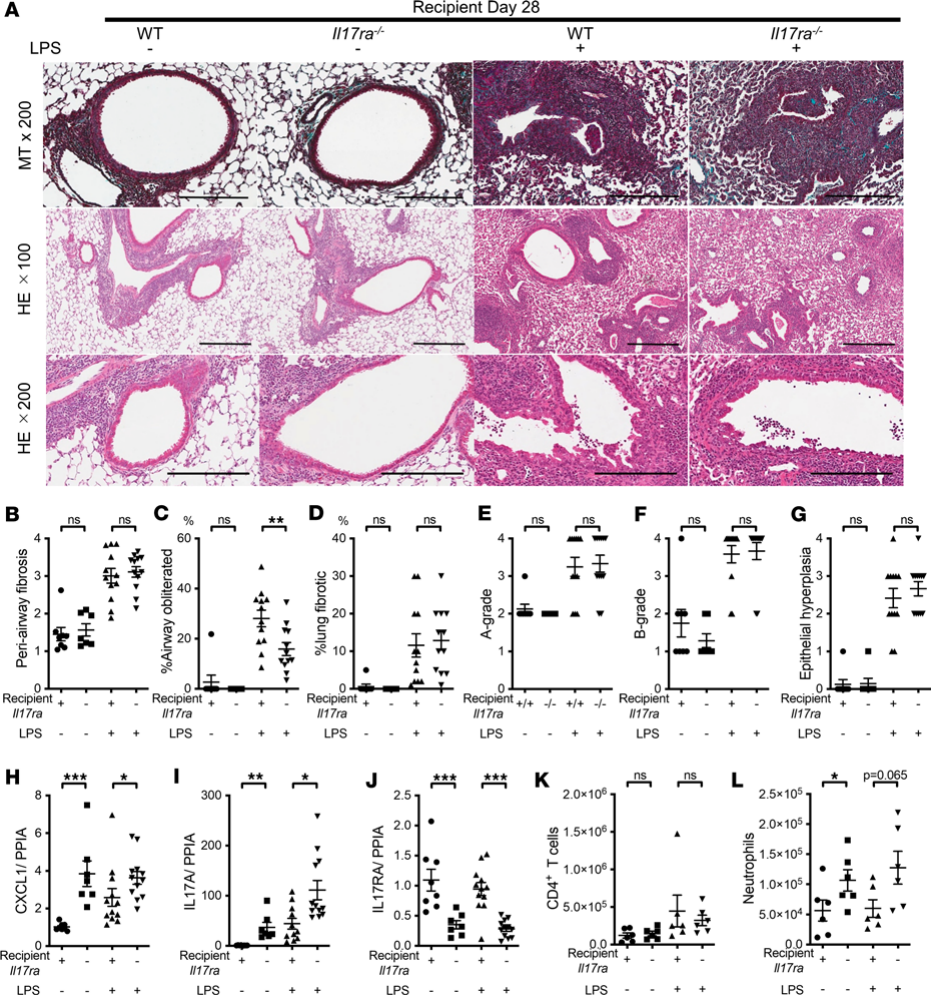
Recipient IL-17RA deficiency reduced airway obliteration with minimal effects on other fibrosis, epithelial change, and inflammation endpoints. The experimental groups are as follows: WT B6 (WT) mice transplanted with B10 lung and *Il17ra^–/–^* mice transplanted with B10 lung under basal condition (*n* = 8 and 7) or under repeated LPS exposures (*n* = 12 each). Under repeated LPS conditions, recipient mice received 6 doses of intratracheal LPS (5 μg in 50 μL PBS) on serial (2/week) postoperative days from day 3 to day 21. The grafts were analyzed on day 28. (**A**) Representative images of the lung grafts. Upper, MT staining (scale bar: 200 μm); middle, H&E staining around airways and vessels (scale bar: 300 μm); bottom, H&E staining showing airways (scale bar: 200 μm). (**B**) Periairway fibrosis score. (**C**) Percentage of obliterated airways. (**D**) Percentage of lung parenchymal fibrosis. (**E**) A-grade according to ISHLT criteria. (**F**) B-grade according to ISHLT criteria. (**G**) Epithelial hyperplasia. Mann-Whitney *U* test. **P < 0.01. (**H**–**J**) CXCL1, IL-17A, and IL-17RA transcripts expression in lung grafts (*n* = 7–12). (**H**) Relative expression of CXCL1 transcripts normalized to PPIA. (**I**) Relative expression of IL-17A transcripts normalized to PPIA. (**J**) Relative expression of IL-17RA transcripts normalized to PPIA. Mann-Whitney *U* test. *P < 0.05; **P < 0.01; ***P < 0.001. (**K** and **L**) Flow cytometry analysis of CD4^+^ T cells and neutrophils in lung allografts (*n* = 6 each). (**K**) The number of CD4^+^ T cells. (**L**) The number of neutrophils. Mann-Whitney *U* test. *P < 0.05.

First, we transplanted B10 lungs into B6 *Il17ra^–/–^* mice versus B6 WT mice. In the absence of repeated airway LPS administrations, WT and *Il17ra^–/–^* recipients showed similar lung histology (Figure 9, A–G). With LPS administrations, compared with WT, *Il17ra^–/–^* recipients did not show significant differences in periairway fibrosis and fibrotic lung area (Figure 9, A, B, and D), acute rejection (Figure 9, E and F), airway hyperplasia (Figure 9G), airway flattening and destruction (Supplemental Figure 5, A and B), or CCSP, TIMP1, or MMP9 transcript levels (Supplemental Figure 5, C–E). The only significant histological difference was decreased obliterated airways in *Il17ra^–/–^* recipient mice compared with WT mice after repeated exposures to LPS (Figure 9C).

*Il17ra^–/–^* recipients showed, as expected, reduced IL-17RA transcript expression both in the absence and presence of repeated airway LPS administration (Figure 9J), but — perhaps through compensatory mechanisms — CXCL1 and IL-17A transcripts were significantly higher (Figure 9, H and I), without a change in IFN-γ transcript level (Supplemental Figure 5F). We also assessed IL-10 transcripts in the grafts since the IL-17A and IL-17A–driven inflammation can be controlled by this antiinflammatory cytokine (58, 59). Levels of IL-10 transcripts trended lower in *Il17ra^–/–^* in the absence of LPS exposure, while they did not differ under LPS exposure (Supplemental Figure 5G). The numbers of total CD4^+^ T cells (Figure 9K) and subsets of CD4^+^ T cells (Supplemental Figure 5, H and I) were not significantly different between the WT and *Il17ra^–/–^* recipients, both in the absence and presence of repeated airway LPS administrations. *Il17ra^–/–^* recipients actually had higher numbers of total neutrophils in the lung grafts than WT recipients, in the absence of LPS, with a similar trend in the presence of LPS (Figure 9L). These results suggest that, in the absence of recipient-derived IL-17RA^+^ leukocytes, compensatory signaling pathways are upregulated, and this may lead to increased leukocyte recruitment through CXCL1 and IL-17A. However, these leukocyte changes did not significantly affect LPS-related chronic lung fibrosis pathology.

### IL-17RA deficiency on donor cells decreases acute rejection histology, fibrosis, and epithelial hyperplasia, without significant effects on inflammatory cells.

**Figure 10:**
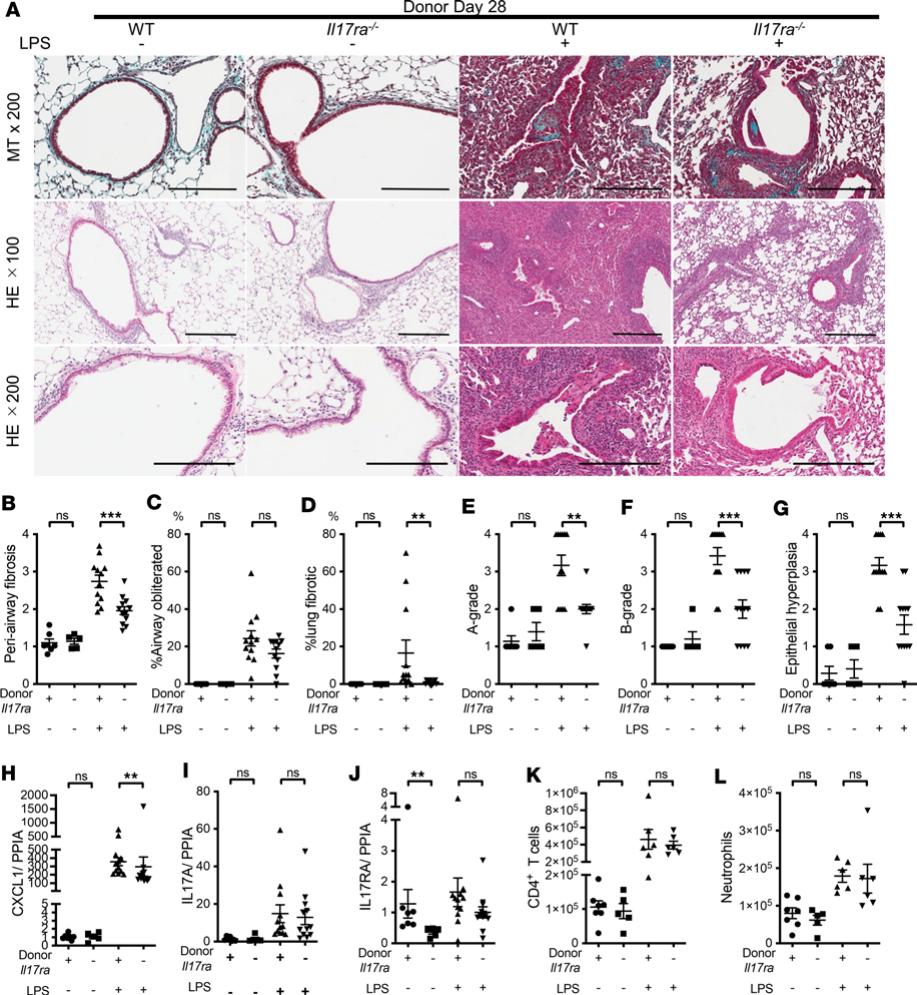
Donor IL-17RA deficiency attenuates allograft acute rejection, epithelial hyperplasia, and airway fibrosis in the setting of repeated LPS exposures. Experimental groups are as follows: B10 mice transplanted with WT lung or *Il17ra^–/–^* lung under basal condition (*n* = 7 and 5, respectively) or under repeated LPS exposures (*n* = 12 each). Under repeated LPS conditions, recipient mice received 6 doses of intratracheal LPS (5 μg in 50 μL PBS) on serial (2/week) postoperative days from day 3 to day 21. The grafts were analyzed on day 28. (**A**) Representative pictures of the lung grafts. Upper, MT staining (scale bar: 200 μm); middle, H&E staining around airways and vessels (scale bar: 300 μm); bottom, H&E staining showing airways (scale bar: 200 μm). (**B**) Periairway fibrosis score. (**C**) Percentage of obliterated airways. (**D**) Percentage of lung parenchymal fibrosis. (**E**) A-grade. (**F**) B-grade. (**G**) Epithelial hyperplasia. Mann-Whitney *U* test. **P < 0.01; ***P < 0.001. (**H**–**J**) CXCL1, IL-17A, and IL-17RA transcripts expression in lung grafts (*n* = 5-12). (**H**) Relative expression of CXCL1 transcripts normalized to PPIA. (**I**) Relative expression of IL-17A transcripts normalized to PPIA. (**J**) Relative expression of IL-17RA transcripts normalized to PPIA. Mann-Whitney *U* test. **P < 0.01. (**K** and **L**) Flow cytometry analysis of CD4^+^ T cells and neutrophils in lung allografts (*n* = 5–7). (**K**) The number of CD4^+^ T cells. (**L**) The number of neutrophils. Mann-Whitney *U* test.

We tested the role of donor IL-17RA in our model in the presence or absence of repeated airway LPS administrations. We transplanted donor lungs from B6 WT or B6 *Il17ra^–/–^* mice to recipient B10 mice. In the absence of LPS, there were no significant differences in histological changes between WT and *Il17ra^–/–^* donor lung allografts (Figure 10, A–G). However, in the presence of repeated airway LPS administrations, *Il17ra^–/–^* donor lung allografts showed significantly attenuated periairway fibrosis and fibrotic lung area (Figure 10, A, B, and D), acute rejection histology (Figure 10, E and F), and epithelial hyperplasia (Figure 10G) compared with WT controls. The proportion of obliterated airways, epithelial destruction, and epithelial hyperplasia were not significantly different (Figure 10C and Supplemental Figure 6, A and B).

IL-17RA transcript expression was reduced in *Il17ra^–/–^* donor lung allografts compared with WT donor lung allografts in the absence LPS administration but not with LPS, presumably because of the higher influx of IL-17RA^+^ recipient immune cells (Figure 10J). Interestingly, CXCL1 transcript expression was significantly attenuated in *Il17ra^–/–^* donor lung allografts under LPS, compared with the WT group (Figure 10H). No significant differences were seen in IL-17A, CCSP, TIMP1, MMP9, and IFN-γ transcript levels (Figure 10I and Supplemental Figure 6, C–F). Compensatory changes of IL-10 levels in the grafts were not observed (Supplemental Figure 6G). Flow cytometry revealed that numbers of CD4^+^ T cells, subsets of CD4^+^ T cells, and neutrophils did not differ between WT and *Il17ra^–/–^* groups (Figure 10, K and L, and Supplemental Figure 6, H and I). These results suggest that, in the absence of LPS, the lack of donor IL-17RA does not significantly affect cellular recruitment or pathology. However, under repeated airway innate immune stimulation, IL-17RA^+^ donor lung allograft cells participate in inflammation and fibrosis.

### IL-17RA staining in human CLAD lungs.

**Figure 11:**
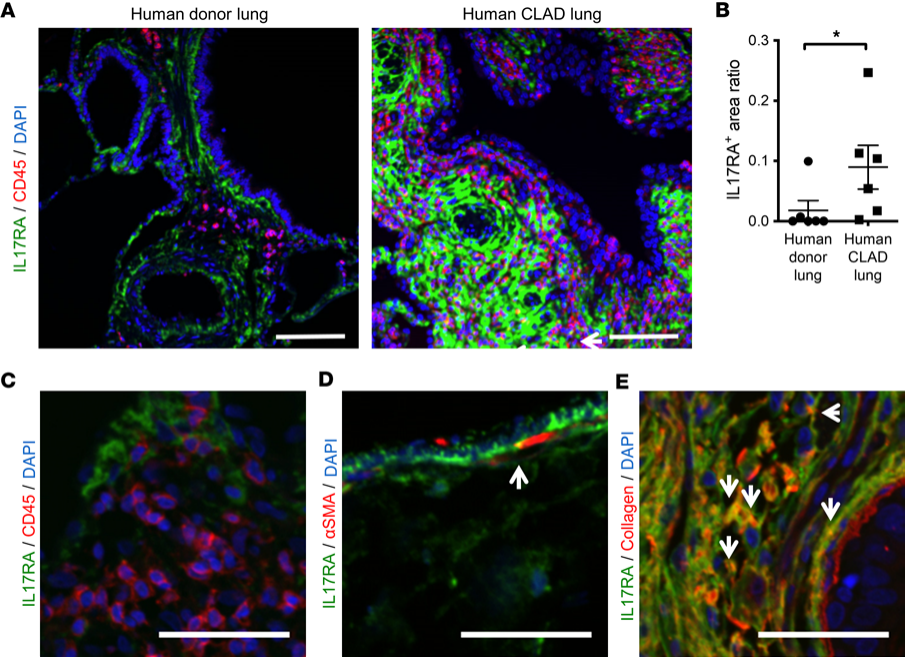
IL-17RA expression in human CLAD lungs. Human CLAD allografts from patients with repeated pulmonary infection episodes after transplant and control donor lungs are shown. (**A**) Representative images of immunofluorescence staining for CD45 and IL-17RA of human explanted CLAD lungs and control lungs. Red, CD45; green, IL-17RA. CLAD lung showed IL-17RA staining around airways and vessels. CD45 staining minimally overlapped with IL-17RA. The arrow indicates that IL-17RA costained with CD45. Scale bar: 100 μm. (**B**) IL-17RA^+^ staining area over lung section area. IL-17RA^+^ area in explanted CLAD lungs was trend toward large compared with control donor lungs (*P* = 0.0519). Mann-Whitney *U* test. (**C**–**E**) Representative pictures of immunofluorescence for IL-17RA, CD45, and α-SMA. (**C**) Representative immunofluorescence of IL-17RA and CD45. Green, IL-17RA; red, CD45; blue, DAPI. Scale bar: 50 μm. (**D**) Representative immunofluorescence of IL-17RA and α-SMA. Scale bar: 50 μm. Arrow indicates IL-17RA and α-SMA double-positive staining. Green, IL-17RA; red: α-SMA; blue, DAPI. (**E**) Representative immunofluorescence of IL-17RA and Collagen. Scale bar: 50 μm. Arrows indicate IL-17RA and Collagen double-positive staining. Green, IL-17RA; red: collagen; blue, DAPI.

IL-17RA immunofluorescence staining in explanted CLAD lungs was enhanced compared with that in human donor lungs (Figure 11, A and B, and Supplemental Figure 7A). Additionally, IL-17RA staining was present around airways (without visible staining of epithelial cells) and vessels and within the fibrotic parenchyma, similar to the staining pattern seen in mouse LPS allografts (Figure 11A). Only a small proportion of IL-17RA^+^ cells costained with CD45 or α-SMA (Figure 11, A, C, and D, and Supplemental Figure 7, B and C). In contrast, many IL-17RA^+^ cells costained with collagen (Figure 11E and Supplemental Figure 7D). These results suggest that structural lung cells, specifically fibroblasts, rather than immune cells, are important expressors of IL-17RA at this late stage of the disease.

## Discussion

In this study, we developed a model of CLAD with airway-predominant lung allograft fibrosis by combining minor alloantigen-mismatched mouse lung transplantation and repeated LPS inhalations. Our results indicate that lung allograft acute rejection and airway fibrosis are driven by repeated episodes of airway innate immune stimulation with involvement of donor IL-17RA^+^ cells.

CLAD has multiple subtypes. Most patients have BOS (3) with airway-predominant fibrosis, and a smaller subset of patients have RAS (4) with diffuse parenchymal fibrosis. We recently reported that minor alloantigen-mismatched mouse lung transplantation with prolonged graft storage and augmented ischemia-reperfusion injury contributes to lung allograft parenchymal fibrosis and airway fibrosis — pathological changes resembling human RAS (20). In contrast, in the present study, we showed that repeated airway LPS stimulation of minor alloantigen-mismatched lung allografts leads primarily to airway obliteration with periairway fibrosis, rather than parenchymal fibrosis. This fibrosis is associated with concurrent augmented cellular infiltration, consistent with acute rejection pathology. These pathological features of fibrosis and increased cellularity seen in the current model are similar to those of human BO. Similar findings were seen in rat lung transplantation with airway LPS administration (22). These findings suggest that subtypes of CLAD may be, at least in part, determined by the types and locations of innate immune stimuli damaging the lung grafts (60).

The lung is not a sterile organ; it is constantly exposed to the environment, to pathogens, and to inhaled innate immune stimuli (61). The airway microbiome affects lung allograft function and has been implicated in airway remodeling (19, 62). These effects may be mediated by pathogen-associated molecular patterns, such as LPS. Lung transplant recipients with polymorphisms in TLR4 and sCD14, which mediate immune responses to LPS, were found to have increased acute rejection and CLAD (63, 64). Consistent with these previous findings (22), our experiments revealed that a limited alloimmune response, in the setting of minor alloantigen mismatch, can be augmented by LPS, leading to acute rejection and fibrosis. Innate immune activation and augmented inflammation may, thus, play a key role in development of CLAD by promoting alloimmunity and preventing healing and resolution of damage.

Airway epithelial injury has been identified as a likely common mechanism resulting in CLAD fibrosis. Airway epithelial abnormalities and damage have been noted in CLAD lungs in humans and mice (17, 65, 66). In addition, airway-centered (or B-grade) acute cellular rejection represents one of the most important risk factors for CLAD (67). In our observations, airway epithelial injury, hyperplasia, and flattening occurred in allografts, and not in syngrafts, exposed to LPS. These changes were similar to those seen in human CLAD lungs (Figure 1). Club cells are specialized airway epithelial cells that play a key role in airway repair and homeostasis. Loss of club cells can contribute to dysregulated repair of airways in lung grafts (68). Indeed, we found reduced expression of CCSP in lung allografts following repeated airway LPS exposures. Similar changes were previously observed in human CLAD lungs (49). We found that both alloimmunity and innate immune activation were required for loss of CCSP and club cells in our model. Loss of club cells in allogeneic lung transplantation was found to recapitulate airway-dominant CLAD-like pathology in another mouse lung transplant model (69). Strategies involving CCSP supplementation and club cell manipulation may provide additional insights into the mechanisms of airway damage–mediated fibrosis in CLAD.

Several human lung transplant studies revealed that neutrophils in the BAL are associated with CLAD (70–72). A recent study by Lendermon et al. has shown that lung transplantation using T-bet^–/–^ mice, which lack Th1 differentiation capability and preferentially upregulate Th17 pathways, resulted in neutrophil predominant lung rejection (57). Besides their well-known effector functions, neutrophils promote rejection after lung transplantation (54, 55). In spite of the evidence that neutrophils are associated with CLAD, it remains unclear whether they are simple bystanders or actually mediate airway remodeling and fibrosis. Our experiments showed somewhat unexpected results. As expected, neutrophils were increased in LPS allografts compared with PBS allografts, likely through cytokines and chemokines, including IL-17A and CXCL1 (73). Paradoxically, *Il17ra^–/–^* recipients had higher numbers of neutrophils together with higher IL-17A transcripts. Gupta et al. also found compensatory increase of IL-17A–producing Th17 cells and γδ T cells in lung allografts subjected to IL-17A blockade (28). We postulate that the compensatory increase in IL-17A in our experiments may have triggered IL-17RA–mediated increases in donor epithelial cytokine expression. Most importantly, we noted a lack of correlation between neutrophil recruitment and fibrosis or acute rejection pathology. The numbers of neutrophils in recipient IL-17RA and donor IL-17RA deficiency on day 28 were not associated with the extent of fibrosis or perivascular/periairway rejection grades. We cannot rule out that neutrophils played a role at earlier time points during the posttransplant course. Neutrophil depletion experiments will be needed to elucidate this conundrum.

While IL-17A primarily promotes neutrophil recruitment to clear bacterial and fungal infection, it can also lead to tissue damage (33, 74). The role of IL-17A in the development of CLAD has been investigated both in mouse models and in patients who have undergone lung transplantation (16, 26, 27, 30, 31, 75). The use of neutralizing IL-17A antibody, pharmacological IL-17A signal inhibition, or IL-17A deficiency reduced CLAD-like pathology, acute rejection, and inflammation in mouse models (16, 30, 31, 76). IL-17A is elevated in patients with CLAD and also in the setting of acute rejection and infection (26, 77). Consistent with these prior studies, our experiments revealed a connection between innate immune activation, IL-17A upregulation, acute rejection, and CLAD.

The targets of IL-17A — i.e., the cells expressing IL-17RA and IL-17RC — have not been well studied in lung transplantation. Polymorphisms of IL-17RA in recipients were found to be associated with the incidence of primary graft dysfunction and CLAD in humans (78, 79). However, the precise role of recipient versus donor IL-17RA have not been studied in a lung transplantation model. Our previous work using an intrapulmonary tracheal transplantation model showed the importance of IL-17RA on recipient cells. However, the analysis was limited by the nature of the model (lacking small-airway and parenchymal analysis) and its technical drawbacks (inability to perform cytokine and flow cytometric analysis) (80). Interestingly, in the present study, *Il17ra^–/–^* recipients exposed to LPS also exhibited attenuation of airway obliteration. This result indicates that IL-17RA on recipient cells may mediate formation of intra-airway granulation tissue. However, the effects of recipient IL-17RA deficiency on other pathological endpoints was minimal.

Importantly, our present study shows that donor IL-17RA is relevant in augmentation of acute rejection, airway and parenchymal fibrosis, and airway epithelial hyperplasia after LPS stimulation. IL-17A has been shown to act directly on both epithelial cells and fibroblasts (81, 82), capable of promoting epithelial-mesenchymal transition (83). IL-17A can also influence fibroblasts to differentiate into myofibroblasts and can stimulate their production of extracellular matrix (84, 85) and tissue remodeling factors such as TIMP1 and MMP9 (86). We postulate that these direct or indirect mechanisms are mediated by donor IL-17RA and are important for lung allograft airway remodeling and fibrosis. This is further supported by the increased IL-17RA immunoreactivity observed within fibroblasts and myofibroblasts in our mouse model and in human CLAD lungs, consistent with previously published IL-17RA analyses in human lung samples (87). We conclude that the fibrosis-relevant targets of excessively secreted IL-17A include donor structural lung cells, likely fibroblasts and myofibroblasts. Cell-specific KO strategies will be needed to confirm this in future experiments.

Our study has several notable limitations. First, our human CLAD patient cohort is meant to illustrate the concept that repeated infections likely trigger sequential injuries and lead to CLAD. However, this cohort cannot prove causation. CLAD is a multifactorial process (36) and other risk factors, highlighted in Supplemental Table 1, may have played a role in CLAD pathogenesis in these patients. Additionally, while we have noted that the BO-related airway changes are similar across CLAD phenotypes, the number of patients in this cohort is too small to fully elucidate differences between BOS and RAS or between distinct stimuli that may have triggered BO development. Second, the controls in our human cohort come from nontransplanted donor lungs, which have not been exposed to alloimmune stimuli. In future studies, one could consider using non-CLAD allograft tissue as an alternate, potentially more relevant, control for CLAD allografts by using transbronchial biopsies from stable lung transplant recipients. Third, our LPS-exposed mouse lung allografts recapitulate only one of the many CLAD triggers that can be seen in humans, in a focused but very reductionist way. Fourth, our 2-strain combinations that assessed the effects of IL-17RA deficiency are not identical. When studying recipient IL-17RA deficiency, we used our usual B10-to-B6 transplants. However, to study donor IL-17RA deficiency, we had to switch the strains to B6-to-B10 transplants, since IL-17RA–deficient mice are commercially available only on the B6 background. B6-to-B10 transplantation shows less acute and chronic rejection pathology compared with B10-to-B6, as indicated in our prior studies (17, 88). While the pathology was still sufficient to show a strong signal in our KO experiments, it is possible that the fibrotic processes differ between the 2 strain combinations.

In summary, in our experimental model, the lung allografts recapitulate features of BO alloimmune-dependent and airway inflammation–dependent fashion, with persistent elevation of IL-17A and CXCL1. Using *Il17ra^–/–^* mice, we demonstrate that donor IL-17RA has a pivotal role in the development of inflammation-related lung allograft fibrosis. Since CLAD is a heterogeneous disease, resulting from alloimmune responses and innate immune stimuli, further analysis of the IL-17A/IL-17RA axis in CLAD subtypes will be an important step to allow personalization of emerging therapies that target this promising pathway.

## Methods

### Mice.

Eight- to 12-week-old male B6 and B10 mice were purchased from The Jackson Laboratory. *Il17ra^–/–^* mice (Taconic B6 background) were generated as previously described (89) and bred in-house. Twelve- to 15-week-old male *Il17ra^–/–^* mice from Amgen Inc. and age-matched WT B6 mice from Taconic were used.

### Mouse orthotopic lung transplantation and intratracheal LPS administration.

Orthotopic lung transplantation was done as previously described (90). To establish a repeated airway inflammation–dependent mouse CLAD model, recipient mice received 8 intratracheal administrations of 5 μg of LPS (*E*. *coli* O111:B4, MilliporeSigma) in 50 μL PBS or 50 μL of PBS from day 3 to day 21 (day 3, 7, 9, 11, 14, 16, 18, and 21). Three experimental groups, syngrafts exposed to LPS (LPS syngrafts), allografts exposed to PBS (PBS allografts), and allografts exposed to LPS (LPS allografts), were analyzed 5, 14, 28, and 42 days after lung transplantation. For lung graft recovery on day 14, the recipient mice did not receive LPS on day 14.

Recipient mice received 6 intratracheal administrations of 5 μg of LPS in 50 μL PBS from day 3 to day 21 (day 3, 7, 10, 14, 17, and 21) in experiments using *Il17ra^–/–^* mice.

### Antibodies and flow cytometry.

The preparation of lung single-cell suspensions was described previously (20). Briefly, after opening the left atrial appendage and cutting the inferior vena cava, lung grafts were gently flushed with 10 mL of normal saline via the pulmonary trunk and were digested with collagenase A and DNase I for 30 minutes in a gentleMACS tissue dissociator (Miltenyi Biotec). The cells were stained with cocktails of the fluorochrome-conjugated monoclonal antibodies; the monoclonal antibodies were obtained from BioLegend, eBioscience, and BD Biosciences (Supplemental Table 2). Fixable Viability Stain 700 or Fixable Viability Stain 620 (BD Biosciences) was used for dead cell staining. Fixation was performed using 1% PFA for staining without intracellular staining. Intracellular staining was performed using BD cytofix/cytoperm kit. Flow cytometry was performed on an LSR II (BD Biosciences). Data were analyzed by FlowJo software (Tree Star Inc.).

### Quantitative PCR.

Total RNA was extracted from lung tissue using the RNeasy mini kit with QIAzol (Qiagen) and converted into cDNA (iScript Select cDNA Synthesis Kit; Bio-Rad). cDNA was amplified using Sso Advanced Universal SYBR Green Supermix (Bio-Rad) in a Bio-Rad CFX384 Tough Real Time PCR Detection system. All primers were designed across introns (Supplemental Table 3). Ct values were determined using a real-time PCR system equipped in CFX384. Change in expression was calculated using the 2^–ΔΔCt^ method normalized to peptidylprolyl isomerase A (PPIA) as a housekeeping gene.

### Grading of rejection, airway obliteration, and lung parenchymal fibrosis in mouse lung grafts.

Lung samples were fixed with 10% neutral buffered formalin at 4°C overnight and processed. Paraffin-embedded samples were sectioned and stained with H&E or Masson’s trichrome. Acute rejection scores were assigned in a blinded manner according to International Society for Heart and Lung Transplantation (ISHLT) criteria, for semiquantitative measurement of perivascular lymphocyte infiltration (A-grade) and airway inflammation (B-score) (91, 92). The percentage of obliterated airways and the percentage of the lung parenchyma affected by parenchymal fibrosis were determined in a blinded fashion as previously described (17). Briefly, the percentage of parenchymal fibrosis area was estimated across the whole sample, graded as 0%–100%. The percentage of obliterated airways was calculated as the total number of obliterated airways divided by the total airways in the section. The periairway fibrosis score was obtained by calculating the mean of periairway fibrosis grades (scores 0–4) of each airway. Epithelial hyperplasia, epithelial damage, and epithelial flattening were graded as 0–4 for each sample (Supplemental Table 4).

The acute lung injury score was scored in a blinded fashion, based on H&E-stained sections, as previously described (93); the scoring criteria included alveolar hemorrhage, vascular congestion, alveolar fibrin, and leukocyte infiltration. These criteria were graded on a 4-level scale of abnormalities: normal appearance and mild, moderate, and severe injury, scored from 0 to 3, respectively. The grades were then added to obtain the overall score.

Collagen green areas in Masson’s trichrome staining were also analyzed by HALO software (Indica Labs) (94), which can automatically detect the color within scanned histology images. The collagen green areas were divided by the total areas of the sections analyzed for each sample to obtain the percent collagen area. Then, in order to normalize the data across multiple experiments with slightly different staining signals, the value of each sample was divided by the average value of the PBS allograft control group of each experiment.

### Immunofluorescence staining.

Immunofluorescence staining was conducted as described (13). Antibodies are listed in Supplemental Table 5. Nucleus staining was performed with DAPI. Images of CD3 and MPO staining were acquired using an LSM 700 confocal microscope (Carl Zeiss). Ten high-power images were randomly acquired from 3 sections per animal, and the numbers of CD3 and MPO cells were counted in a blinded manner. Images of CCSP and α-SMA staining were acquired using a whole slide system (Zeiss AxioScan; Carl Zeiss). Lung section areas and CCSP^+^ staining areas were analyzed by HALO software.

Dual staining images of IL-17RA and α-SMA, or IL17RA and CD45, in mouse lungs were acquired using a whole slide system. Lung section areas and IL-17RA^+^ staining areas were analyzed by HALO software. Dual staining images of IL-17RA and Collagen I, IL-17RA and Collagen I, IL-17RA and LYVE1, and IL-17RA and CD31 in mouse lungs were acquired using a spinning disk confocal system (WaveFX; Yokogawa Electric Corp.).

Images of IL-17RA and CD45 in human lungs for quantification were acquired using a whole-slide system. Lung section areas and IL-17RA^+^ staining areas were analyzed by HALO software. Dual staining images of IL-17RA and CD45, IL-17RA and α-SMA, and IL-17RA and Collagen I in human lungs were acquired using a spinning disk confocal system.

### μCT.

μCT was performed on days 3, 28, and 42 on a GE μCT device (GE Locus Ultra Micro-CT; GE HealthCare) as previously described (31). Day 3 CT scans were taken before LPS or PBS administration. Segmentation of individual lungs/lobes was performed on a dedicated 3D postprocessing workstation (Vitrea; Vital Images). Longitudinal changes in lung volume were calculated as a ratio of the volume of the left lung to the entire pulmonary volume (i.e., right lung + accessory lobe + left lung). Average lung density was also measured and compared longitudinally (20).

### Human explanted CLAD lungs.

A retrospective review was done of the medical records of patients who underwent retransplantation at the Toronto Lung Transplant Program from June 1, 2016, to February 28, 2019. Patients who had multiple episodes of airway infection, including viral, fungal, and bacterial infections, between first transplantation and second transplantation were selected for this study. Samples of explanted CLAD lungs were obtained at the time of redo transplantation surgery. Control lung samples were procured when donor lung volume reduction was required during lung transplantation. Samples were fixed with 10% neutral buffered formalin at 4°C overnight and processed. Paraffin-embedded samples were sectioned and stained with H&E or Elastic Trichrome stains.

### Statistics.

Prism 9 (GraphPad Software Inc.) was used for all statistical analyses. Data are expressed as individual points and means or as mean ± SEM. The 2-tailed *t* test, Mann-Whitney *U* test, Kruskal-Wallis test, or 2-way ANOVA were used for the statistical analysis. *P* < 0.05 was deemed significant.

### Study approval.

All animals received care in compliance with the *Guide for the Care and Use of Laboratory Animal*s (National Academies Press, 2011). The experimental protocol was approved by the Animal Care Committee of the Toronto General Research Institute, University Health Network. The CLAD lung samples were collected from patients at the time of redo transplantation. The study protocol was approved by the Institutional Research Ethics Board (no. 15-9531-AE) at the University Health Network.

### Data availability.

The data that support the findings of this study are available from the corresponding author upon request. Values for all data points in graphs are reported in the Supporting Data Values file.

## Author contributions

Project design was contributed by TW, SCJ, and TM. Experiments were conducted by TW, SCJ, KB, TD, CK, GT, ZG, and TM. Data analysis and interpretation were contributed by TW, SCJ, WZ, KB, GT, and TM. Patient cohort design and phenotyping were contributed by GB and JH. CT scan analysis was contributed by MH and TW. Pathology review was contributed by DMH and TM. Project supervision was contributed by SCJ, SK, and TM. Critical contribution to project design and data interpretation were contributed by ML and SK. Manuscript writing was contributed by TW, SCJ, and TM. Manuscript editing was contributed by TW, SCJ, and TM.

## Supplementary Material

Supplemental data

Supporting data values

## Version 1 11/08/2023

Electronic publication
