# Supplementary material for: Donor IL-17 receptor A regulates LPS-potentiated acute and chronic murine lung allograft rejection
Source: JCI Insight. 2023 Nov 8;8(21):e158002. doi: 10.1172/jci.insight.158002 (PMC10721268; doi:10.1172/jci.insight.158002)
Supplement: Supplemental data [file jciinsight-8-158002-s096.pdf]

Supplemental Figure S1

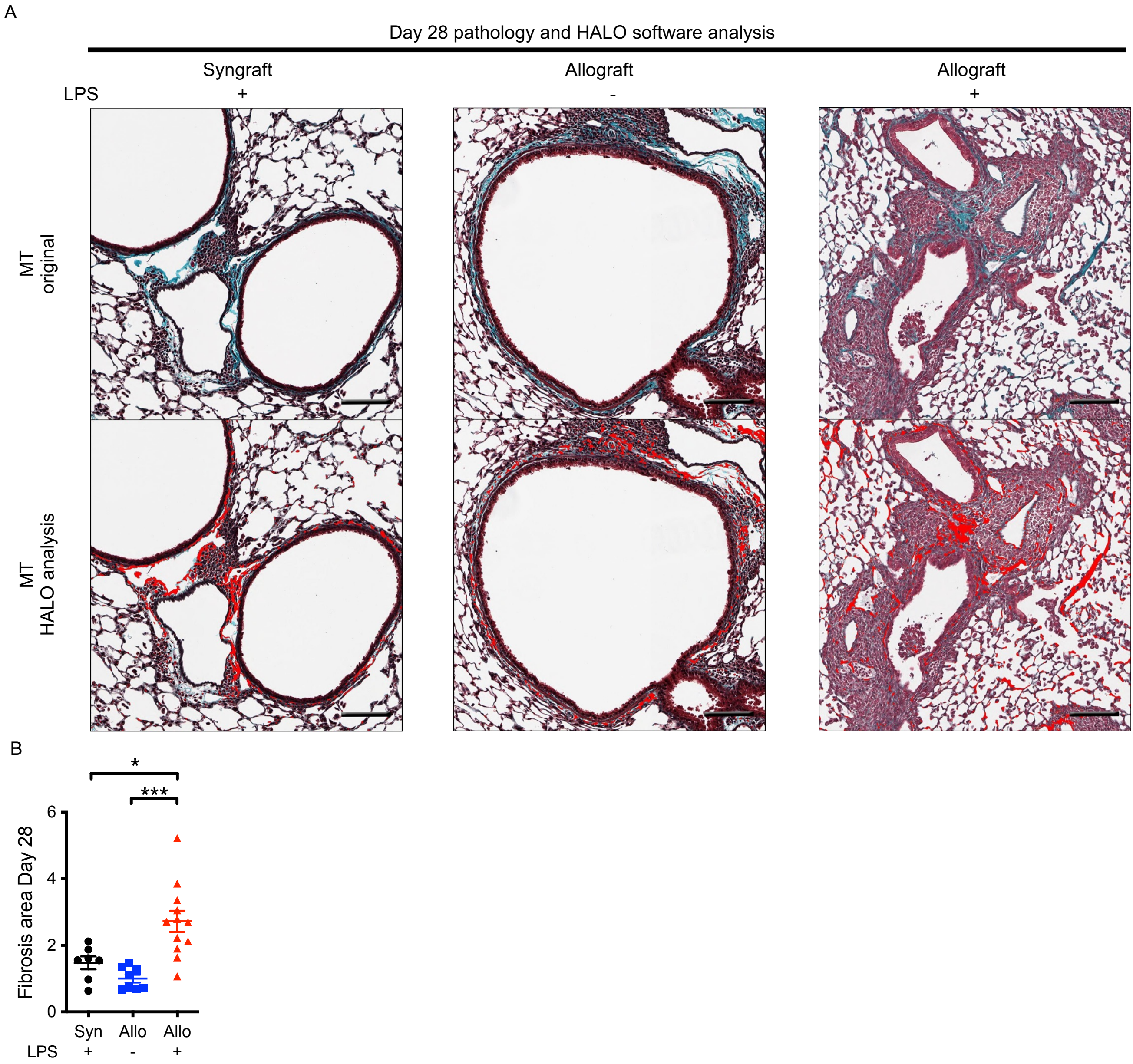

Supplemental Figure S1

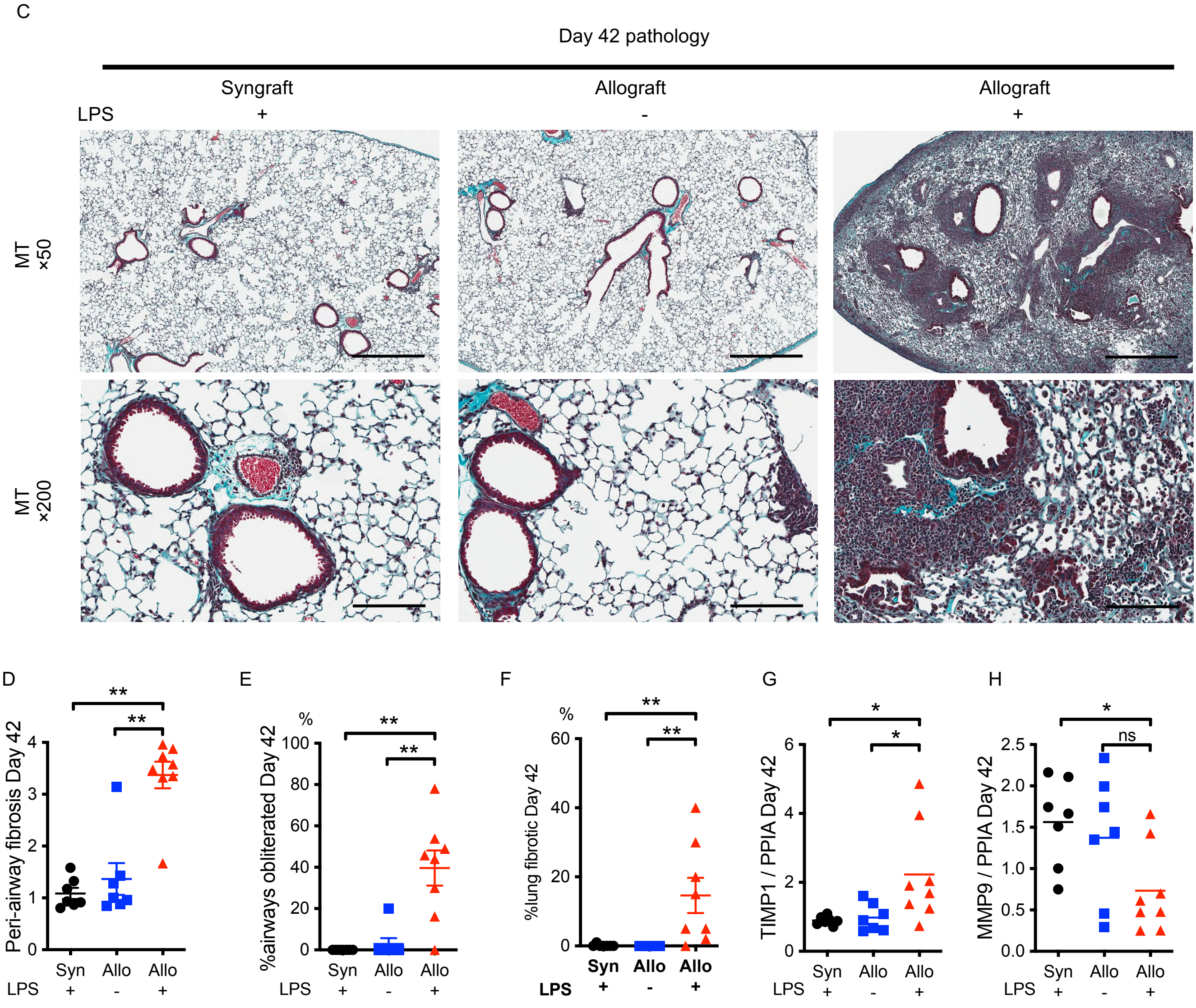

Supplemental Figure S1. Chronic rejection pathology quantification at day 28 and 42.

B6 recipient mice received a single left lung transplant from B6 or B10 donor mice, followed by repeated intra-tracheal LPS vs. PBS exposures. (A) Representative images of graft MT staining on day 28 after lung transplantation used for HALO software analysis. Upper pictures show MT staining imaged by light microscopy. Lower pictures show green fibrotic areas recognized and identified in red by HALO software. Left: LPS-syngrafts. Middle: PBS-allograft. Right: LPS-allograft. scale bar: 100μm. (B) Fibrotic green areas identified by HALO software normalized to the average %fibrotic area of PBS-allograft (n=7-12).

(C) Representative images of MT staining on day 42 after lung transplantation. Scale bar: 200μm. Left: LPS-syngrafts. Middle: PBS-allograft. Right: LPS-allograft. LPS-syngrafts and PBS-allografts showed no obvious fibrosis. LPS-allografts showed augmented peri-airway fibrosis and obliterated airways. (D, E, and F) Fibrotic change scores of the lung grafts (*n*=7 and 8 per group). (D) Peri-airway fibrosis scores. (E) Percentage of obliterated airways. (F) Percentage of lung fibrosis grading. (G) TIMP1 transcript normalized to PPIA in the grafts at day 42. (H) Lung graft MMP9 transcript normalized to PPIA at day 42. Kruskal-Wallis test. **$p < 0.01$; ***$p < 0.001$.

Supplemental Figure S2

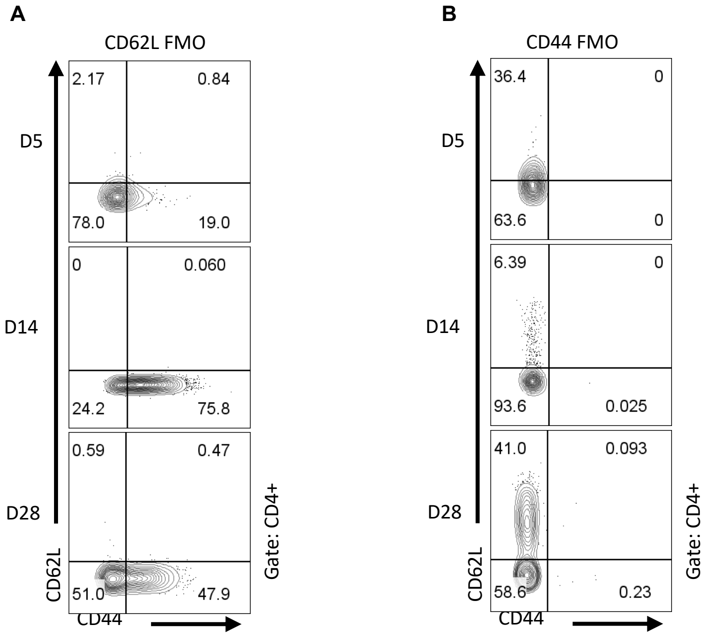

Supplemental Figure S2. Plots showing fluorescence-minus-one controls for CD62L and CD44.

B6 recipient mice received a single left lung transplant from B6 or B10 donor mice, followed by repeated intra-tracheal LPS vs. PBS exposures. Samples were obtained from LPS-syngraft, PBS-allograft, and LPS-allograft on days 5, 14, and 28. Lung graft cells were subjected to flow cytometry. $CD4^{+}$ cells were gated. FMO, fluorescence-minus-one controls for CD62L and CD44 are shown for each timepoint. (A) CD62L. (B) CD44.

Supplemental Figure S3

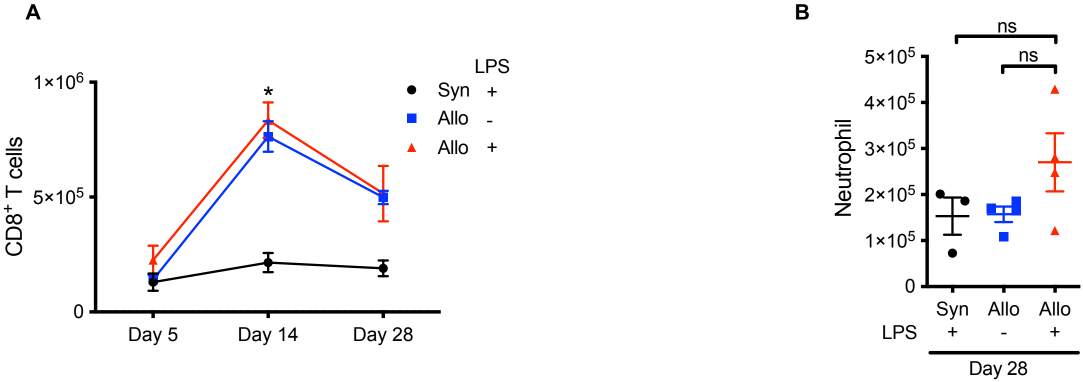

Supplemental Figure S3. Additional flow cytometry plots showing CD8 T cells and neutrophils in mouse syngrafts and allografts under LPS stimulation.

B6 recipient mice received a single left lung transplant from B6 or B10 donor mice, followed by repeated intra-tracheal LPS vs. PBS exposures. (A) The number of $CD8^+$ T cells in the lung grafts. Kruskal-Wallis test. LPS-syngrafts vs LPS-allografts, *$p<0.05$. (B) The number of neutrophils in the lung graft on day 28. Kruskal-Wallis test.

Supplemental Figure S4

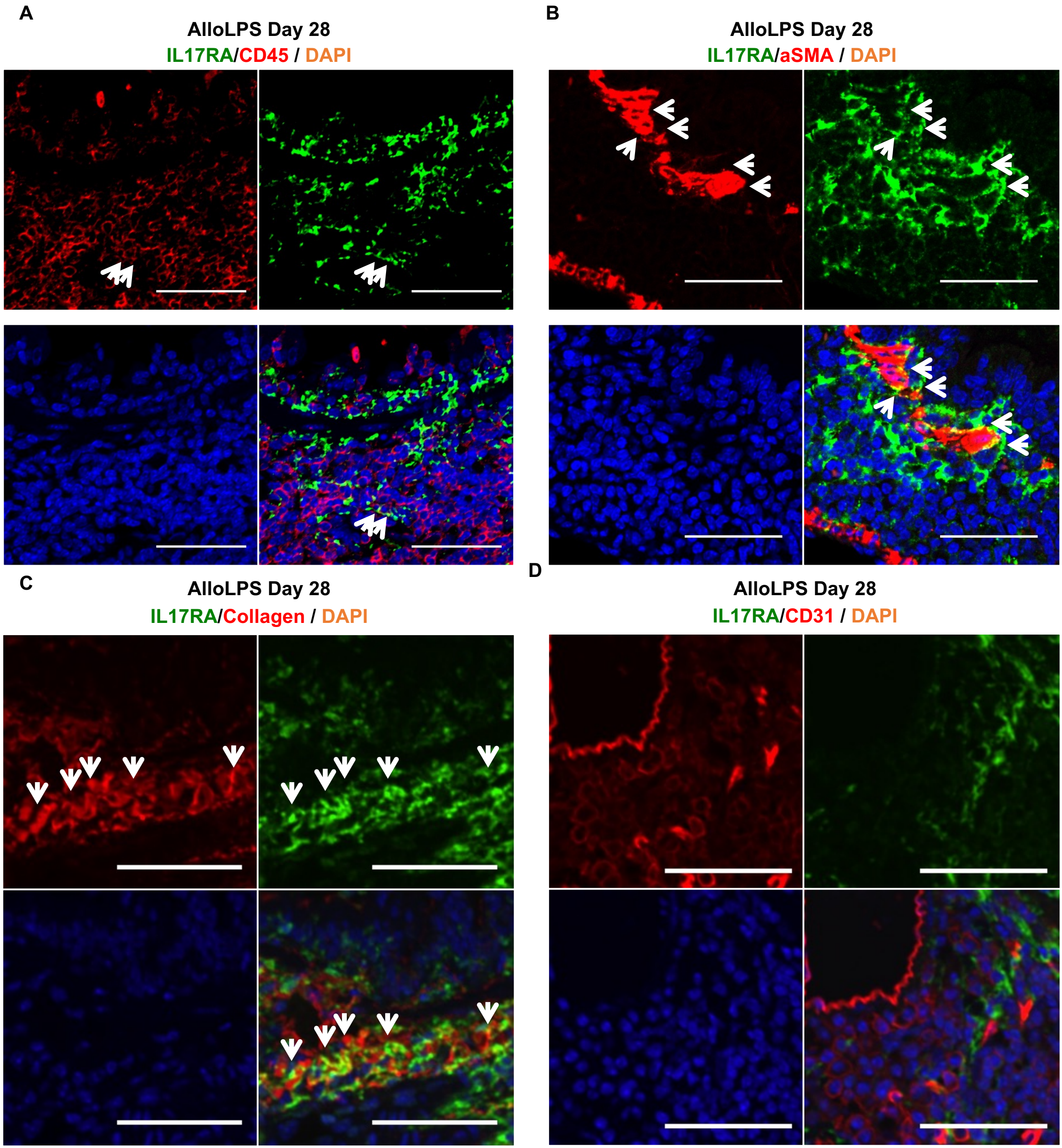

E

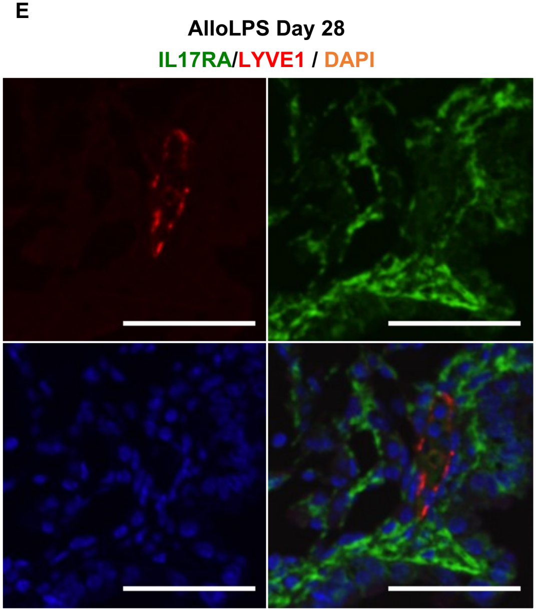

Supplemental Figure S4. Immunofluorescence of mouse lung grafts on day 28

B6 recipient mice received a single left lung transplant from B6 or B10 donor mice, followed by repeated intra-tracheal LPS vs. PBS exposures. Samples were obtained on day 28. Representative pictures of immunofluorescence of lung grafts are shown. The merged pictures are also shown in Figure 8E. (A) Immunofluorescence of the lung grafts for IL17RA and CD45. Green: IL17RA, Red: CD45, Blue: DAPI. Scale bar: 50μm. Arrows indicate CD45 and IL17RA double-positive staining. (B) Immunofluorescence of the lung grafts for IL17RA and aSMA. Green: IL17RA, Red: aSMA, Blue, DAPI. Scale bar: 50μm. Arrows indicate IL17RA and aSMA double-positive staining. (C) Immunofluorescence of the lung grafts for IL17RA and Collagen. Green: IL17RA, Red: collagen, Blue: DAPI. Scale bar: 50μm. Arrows indicate IL17RA and Collagen double-positive staining. (D) Immunofluorescence of the lung grafts for IL17RA and CD31. Green: IL17RA, Red: collagen, Blue: DAPI. Scale bar: 50μm. (E) Immunofluorescence of the lung grafts for IL17RA and LYVE1. Green: IL17RA, Red: LYVE1, Blue: DAPI. Scale bar: 50μm.

Supplemental Figure S5

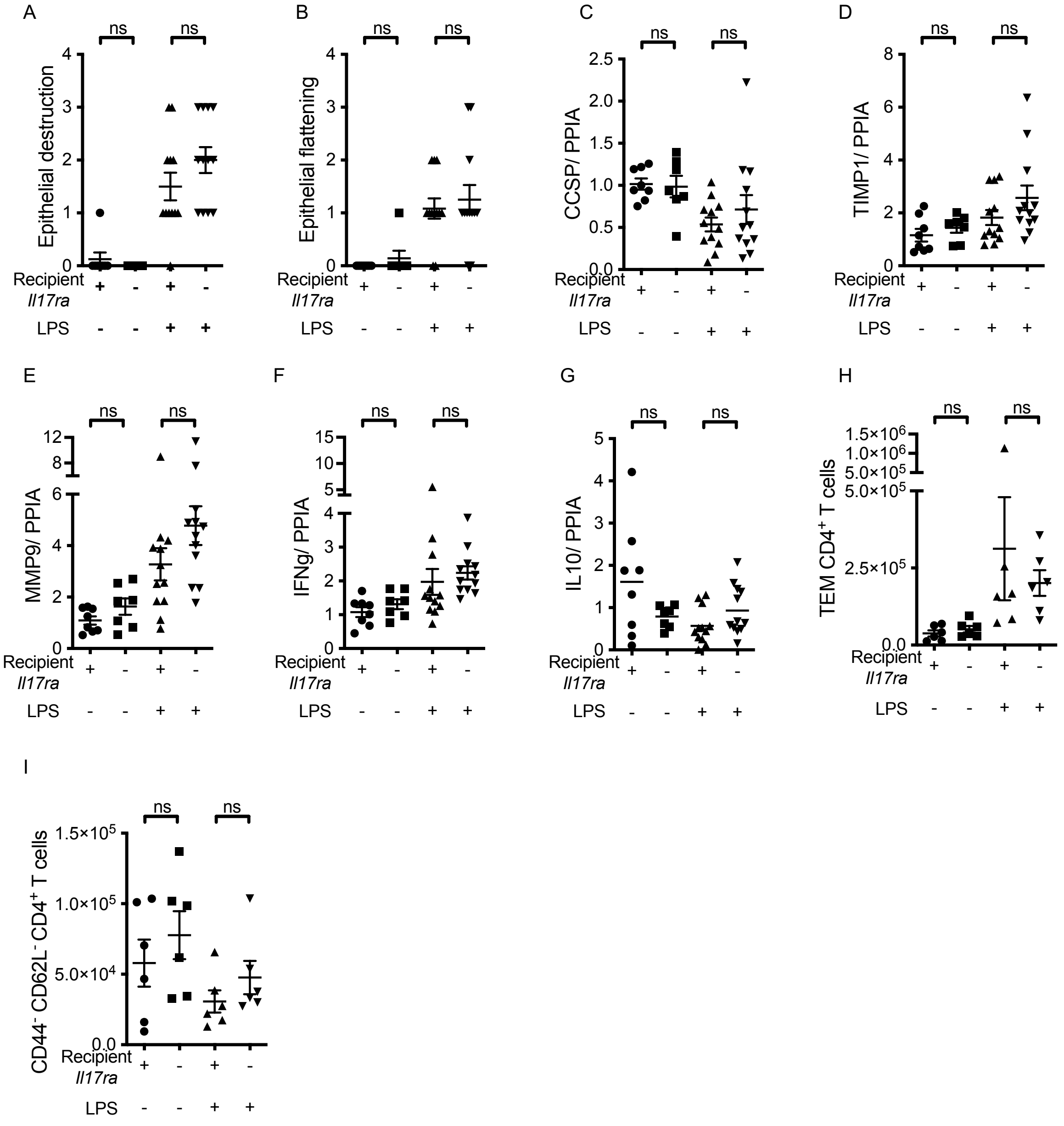

Supplemental Figure S5. Epithelial changes, levels of CCSP, TIMP1, IFNg, and IL10 transcripts, and $CD4^+$ T cell subsets in IL17RA-deficient lung transplant recipient mice

The experimental groups are as follows: Wild type B6 (WT) mice transplanted with B10 lung and *Il17ra*$^{-/-}$ mice transplanted with B10 lung under basal condition (n=8 or 7) or under repeated LPS exposures (n=12 each). Under repeated LPS conditions, recipient mice received 6 doses of intra-tracheal LPS (5μg in 50μl PBS) on serial (2/week) postoperative days from day 3 to day 21. The grafts were analyzed on day 28. (A and B) Airway epithelial scores in the lung grafts on day 28 (n=7-12 per group). (A) Epithelial destruction scores. (B) Epithelial flattening scores. (C-G) Transcript levels of CCSP, TIMP1, MMP9, IFNg, and IL10 in lung allografts on day 28 (n=7-12 per group). (C) Relative expression of CCSP transcripts normalized to PPIA. (D) Relative expression of TIMP1 transcripts normalized to PPIA. (E) Relative expression of MMP9 transcripts normalized to PPIA. (F) Relative expression of IFNg transcripts normalized to PPIA. (G) Relative expression of IL10 transcripts normalized to PPIA. (H and I) Subsets of $CD4^+$ T cells in lung allografts assessed by lung cell flow cytometry on day 28 (n=6 each). (H) Effector memory $CD4^+$ T cells ($CD44^+CD62L^-CD4^+$ T cells, TEM). (I) $CD44^-CD62L^-CD4^+$ T cells. Mann-Whitney test. ***$p < 0.001$.

Supplemental Figure S5

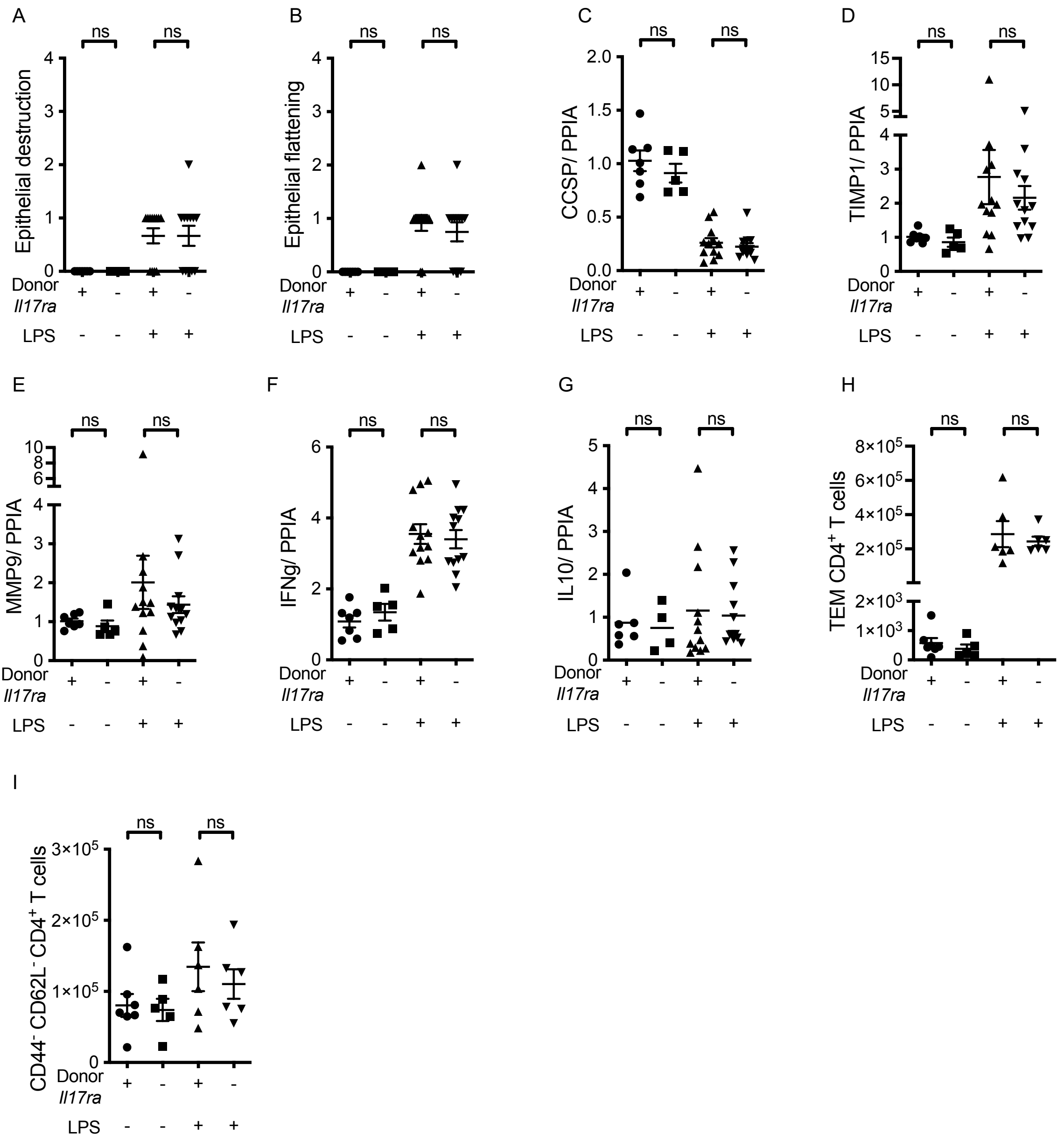

Supplemental Figure S6. Epithelial damage, levels of CCSP, TIMP1, MMP9, IFNg, and IL10 transcripts, and CD4^{+} T cell subsets in IL17RA-deficient donor lung allografts

Experimental groups are as follows: B10 mice transplanted with WT lung or *Il17ra*$^{-/-}$ lung under basal condition (n=7 or 5) or under repeated LPS exposures (n=12 each). Under repeated LPS conditions, recipient mice received 6 doses of intra-tracheal LPS (5μg in 50μl PBS) on serial (2/week) postoperative days from day 3 to day 21. The grafts were analyzed on day 28. (A and B) Airway epithelial scores in the lung grafts on day 28 (n=5-12 per group). (A) Epithelial destruction scores. (B) Epithelial flattening scores. (C-G) Transcript levels of CCSP, TIMP1, MMP9, IFNg, and IL10 in the lung allografts on day 28 (n=5-12 per group). (C) Relative expression of CCSP transcripts normalized to PPIA. (D) Relative expression of TIMP1 transcripts normalized to PPIA. (E) Relative expression of MMP9 transcripts normalized to PPIA. (F) Relative expression of IFNg transcripts normalized to PPIA. (G) Relative expression of IL10 transcripts normalized to PPIA. (H and I) Subsets of CD4^{+} T cells in lung allografts assessed by lung cell flow cytometry on day 28 (n=5-7 per group). (H) Effector memory CD4^{+} T cells (CD44^{+}CD62L^{-}CD4^{+} T cells, TEM). (I) CD44^{-}CD62L^{-}CD4^{+} T cells. Mann-Whitney test.

Supplemental Table 1: Clinical data of re-transplant recipients

	Case1	Case 2	Case 3	Case 4	Case 5	Case 6
Age: at re-Tx	50	36	24	29	59	34
Gender	M	F	F	M	F	M
Primary disease	COPD	BPD	Cystic Fibrosis	Cystic Fibrosis	Pulmonary Fibrosis	Pulmonary Fibrosis
IS baseline	csa/aza/pred	csa/mmf/pred	csa/mmf/pred	csa/aza/pred	csa/aza/pred	csa/aza/pred
IS at re-Tx	tac/mmf/pred	tac/mmf/pred	tac/mmf/pred	tac/mmf/pred	tac/mmf/pred	tac/mmf/pred
HLA mismatch (A/B/C/DR/DQ)	7/10	7/10	6/10	6/10	7/10	9/10
1st dnDSA	DQ	DQA5-DQB2	B8, DQ9	none	DQA5	DRw53, DQ7, DQA3
Time to 1st dnDSA, days	84	32	433	N/A	94	86
CMV status	D-/R+	D-/R-	D+/R+	D-/R-	D+/R+	D+/R+
Pulmonary pathogens [a]	**Haemophilus**	**Haemophilus,** Aspergillus, Mycobacterium Avium	**Pseudomonas**, Staphylococcus	**Haemophilus**, Aspergillus, Influenza B virus **Pseudomonas**	**Escherichia coli**, Staphylococcus, Aspergillus, CMV, RSV	**Mycoplasma**, **Pseudomonas,** Aspergillus, CMV, RSV, Mycobacterium fortuitum
Steroid pulses before CLAD onset, n	0	1	1	2	0	0
Steroid pulses after CLAD onset, n	0	0	1	0	0	0
ATG before CLAD onset, n	0	2	0	0	1	0
ATG after CLAD onset, n	1	0	1	1	0	0
Total reflux [b]	31	12	27	18	35	N/A
CLAD phenotype [c]	BOS	Mixed	RAS	RAS	Undefined	BOS
Time to CLAD, days	170	779	472	250	715	254
Time from CLAD to re-Tx, days	334	391	310	218	74	386
Sum of A[d] grades	0	1	3	1	1	0
Sum of B grades[d]	0	0	0	0	0	0

Legend:
Abbreviations: re-Tx, re-transplant; COPD, Chronic obstructive pulmonary disease; BPD, Bronchopulmonary Dysplasia; IS, immunosuppression; csa, cyclosporine A; aza, azathioprine; pred, prednisolone; mmf, mycophenolate mofetil; tac, tacrolimus; HLA, human leukocyte antigen: dnDSA, de novo donor-specific antibody: CMV, Cytomegalovirus; RSV, Respiratory syncytial virus; CLAD, chronic lung allograft dysfunction; ATG, anti-thymocyte globulin; BOS, bronchiolitis obliterans syndrome; RAS, restrictive allograft syndrome.
a: Microorganisms identified between first transplant and re-transplant are listed. Gram-negative pathogens are bolded. b: Number of reflux episodes as tested by 24-hour pH-impedance probe per protocol around 3 months post-transplant. 48 episodes in 24 hours were considered as clinically diagnostic of gastro-esophageal reflux by our clinical laboratory. c: CLAD phenotypes at time of CLAD onset were determined per the 2019 ISHLT consensus guidelines. D: Sums of A or B grades are based on the ISHLT A and B histological acute rejection grading of transbronchial biopsies obtained between transplant and re-Tx. Detailed grades are shown in figures 1A-F.

Supplemental Table 2: Antibodies for flow cytometery

Companey	Fluorochrome	Antigen	Catalog number	Clone
BioLegend	PE	CD49b	103506	HMa2
	BV510	CD4	100449	GK1.5
	BV605	CD45	103140	30-F11
	BV650	CD19	115541	6D5
	BV650	CD86	105036	GL-1
	PerCP-Cy5.5	CD44	103032	IM7
	PE-Cy7	TCRb	109222	H57-597
eBioscience	FITC	CD3	11-0032-82	17A2
	APC	IL17RA	17-7182-82	PAJ-17R
	BV421	Ly6g	562737	1A8
	eFluor 660	IL17A	50-7177-82	eBio17B7
	eFluor 780	CD8	47-0081-82	53-6.7
	PerCP-Cy5.5	IFNg	45-7311-82	XMG1.2
BD Biosciences	PE-CF594	CD62L	562404	MEL-14
	PE-CF594	IL4	562450	11B11

Supplemental Table 3: Primer pairs

Transcript	Forward	Reverse
Mouse PPIA	GGTCAACCCCACCGTGTT	GCTCGAAAGTTTTCTGCTGTCT
Mouse IL17A	CCTGGACTCTCCACCGCAA	TTCCCTCCGCATTGACACAG
Mouse IFNg	GCAACAGCAAGGCGAAAAAG	CTCATTGAATGCTTGGCGCT
Mouse CCSP	CAGCTCAGCTTCTTCGGAC	TGGTCTCTTGTGGGAGGGTA
Mouse CXCL1	ACCGAAGTCATAGCCACACTC	CTCCGTTACTTGGGGACACC
Mouse IL17RA	CCTCATCACACTCATCGCCA	GCCGAGTAGACGATCCAGAC
Mouse TIMP1	TCGGACCTGGTCATAAGGGC	GCTTTCCATGACTGGGGTGT
Mouse MMP9	CTCTGCTGCCCCTTACCAG	AGCGGTACAAGTATGCCTCTGC
Mouse IL10	AGAGCAAGGCAGTGGAGCAG	TGGCCTTGTAGACACCTTGGTC

Supplemental Table 4: Histological grading system

Histologic feature	Score	Severity	Features
Peri-Airway Fibrosis (scored per airway)<br>Measure: Average score for each sample.	0	None	No fibrous tissue
	1	Minimal	Fibrous tissue thickness = 1 mononuclear cell
	2	Mild	Fibrous tissue thickness = 2 mononuclear cells
	3	Moderate	Fibrous tissue thickness = 3 mononuclear cells
	4	Severe	Fibrous tissue thickness ≥ 4 mononuclear cells
% airway obliterated	Percent affected		Calculate as the total number of obliterated airways divided by the total airways in the section.
% lung fibrotic	Percent affected		Estimate the percentage of the lung parenchyma affected by fibrosis.
Epithelial hyperplasia (=increased height of columnar epithelial cells, cell crowding, prominent secretory granules/mucin)	0	None	Normal epithelium throughout
	1	Minimal	An occasional airway with epithelial hyperplasia
	2	Mild	Approximately 10% of airways with epithelial hyperplasia
	3	Moderate	Approximately 25% of airways with epithelial hyperplasia
	4	Severe	>25% of airways with epithelial hyperplasia
Epithelial flattening	0	None	Normal epithelium
	1	Minimal	An occasional airway with flattened epithelium
	2	Mild	Approximately 10% of airways with flattened epithelium
	3	Moderate	Approximately 25% of airways with flattened epithelium
	4	Severe	>25% of airways with flattened epithelium
Epithelial destruction	0	None	Normal epithelium
	1	Minimal	An occasional airway with destructed epithelium
	2	Mild	Approximately 10% of airways with destructed epithelium
	3	Moderate	Approximately 25% of airways with destructed epithelium
	4	Severe	>25% of airways with destructed epithelium

Supplemental Table 5: Antibodies for immunofluorescence

Immunofluorescence for CD3 and MPO			
Primary antibodies		Secondary antibodies	
anti-CD3 antibody	ab11089, Abcam, Toronto, ON	Goat anti-rat IgG, Alexa 555	Thermo Fisher Scientific, Mississauga, ON
anti-MPO antibody	Ab9535, Abcam	Donkey anti-rabbit IgG, Alexa 488	Thermo Fisher Scientific
**Immunofluorescence for CCSP and aSMA**			
Primary antibodies		Secondary antibodies	
anti-CCSP antibody	sc-9772, Santa Cruze, CA	Donkey anti-rabbit IgG, Alexa 488	Thermo Fisher Scientific
Cy3 conjugated anti-aSMA antibody	C6198, Sigma	N/A	N/A
**Immunofluorescence for IL17RA and αSMA in mouse lung**			
Primary antibodies		Secondary antibodies	
anti-IL17RA antibody	ab218249, Abcam	Alexa 488 SuperBoost™ tyramide kit	Thermo Fisher Scientific
Cy3 conjugated anti-aSMA antibody	C6198, Sigma	N/A	N/A
**Immunofluorescence for IL17RA and CD45 in mouse lung**			
Primary antibodies		Secondary antibodies	
anti-IL17RA antibody	ab218249, Abcam	Alexa 488 SuperBoost™ tyramide kit	Thermo Fisher Scientific
anti-CD45 antibody	ab10558, Abcam	Donkey anti-rabbit IgG, Alexa 555	Thermo Fisher Scientific
**Immunofluorescence for IL17RA and Collagen in mouse lung**			
Primary antibodies		Secondary antibodies	
anti-IL17RA antibody	ab218249, Abcam	Alexa 488 SuperBoost™ tyramide kit	Thermo Fisher Scientific
anti-Collagen I antibody	ab34710, Abcam	Donkey anti-rabbit IgG, Alexa 555	Thermo Fisher Scientific
**Immunofluorescence for IL17RA and CD31 in mouse lung**			

Primary antibodies		Secondary antibodies	
anti-IL17RA antibody	ab218249, Abcam	Alexa 488 SuperBoost™ tyramide kit	Thermo Fisher Scientific
anti-CD31 antibody	AF3628, Novus, Littleton, CO	Donkey anti-rabbit IgG, Alexa 555	Thermo Fisher Scientific
Immunofluorescence for IL17RA and LYVE1 in mouse lung			
Primary antibodies		Secondary antibodies	
anti-IL17RA antibody	ab218249, Abcam	Alexa 488 SuperBoost™ tyramide kit	Thermo Fisher Scientific
anti-LYVE1 antibody	NB110-61026, Novus	Donkey anti-rabbit IgG, Alexa 555	Thermo Fisher Scientific
Immunofluorescence for IL17RA and LYVE1 in mouse lung			
Primary antibodies		Secondary antibodies	
anti-IL17RA antibody	ab218249, Abcam	Alexa 488 SuperBoost™ tyramide kit	Thermo Fisher Scientific
anti-LYVE1 antibody	NB110-61026, Novus	Gaot anti-rabbit IgG, Alexa 555	Thermo Fisher Scientific
Immunofluorescence for IL17RA and CD45 in human lung for quantification			
Primary antibodies		Secondary antibodies	
anti-IL17RA antibody	ab218249, Abcam	Alexa 488 SuperBoost™ tyramide kit	Thermo Fisher Scientific
anti-CD45 antibody	ab10558, Abcam	Donkey anti-rabbit IgG, Alexa 555	Thermo Fisher Scientific
Immunofluorescence for IL17RA and αSMA in human lung for representative image			
Primary antibodies		Secondary antibodies	
anti-IL17RA antibody	ab180904, Abcam	Alexa 488 SuperBoost™ tyramide kit	Thermo Fisher Scientific
Cy3 conjugated anti-aSMA antibody	C6198, Sigma	N/A	N/A
Immunofluorescence for IL17RA and CD45 in human lung for representative image			
Primary antibodies		Secondary antibodies	
anti-IL17RA antibody	ab180904, Abcam	Alexa 488 SuperBoost™ tyramide kit	Thermo Fisher Scientific

anti-CD45 antibody	ab10558, Abcam	Donkey anti-rabbit IgG, Alexa 555	Thermo Fisher Scientific
Immunofluorescence for IL17RA and Collagen in human lung for representative image			
Primary antibodies		Secondary antibodies	
anti-IL17RA antibody	ab180904, Abcam	Alexa 488 SuperBoost™ tyramide kit	Thermo Fisher Scientific
anti-Collagen I antibody	ab233080, Abcam	Donkey anti-rabbit IgG, Alexa 555	Thermo Fisher Scientific
